# A New Insight into the Mechanism of Atrazine-Induced Neurotoxicity: Triggering Neural Stem Cell Senescence by Activating the Integrated Stress Response Pathway

**DOI:** 10.34133/research.0547

**Published:** 2024-12-13

**Authors:** Jian Chen, Xue-Yan Dai, Kanwar K. Malhi, Xiang-Wen Xu, Yi-Xi Tang, Xiao-Wei Li, Jin-Long Li

**Affiliations:** ^1^ College of Veterinary Medicine, Northeast Agricultural University, Harbin 150030, P.R. China.; ^2^ Jiangxi Provincial Key Laboratory for Animal Health, Institute of Animal Population Health, College of Animal Science and Technology, Jiangxi Agricultural University, Nanchang 330045, P.R. China.; ^3^ Key Laboratory of the Provincial Education Department of Heilongjiang for Common Animal Disease Prevention and Treatment, Northeast Agricultural University, Harbin 150030, P.R. China.

## Abstract

Atrazine (AT), a widely utilized chemical herbicide, causes widespread contamination of agricultural water bodies. Recently, exposure to AT has been linked to the development of age-related neurodegenerative diseases (NDs), suggesting its neurotoxicity potential. As an endocrine disruptor, AT targets the hypothalamus, a crucial part of the neuroendocrine system. However, the toxicological mechanism of AT exposure to the hypothalamus and its correlation with ND development remain unexplored. Our results indicated that AT exposure caused significant morphological and structural damage to the hypothalamus, leading to the loss of mature and intact neurons and microglial activation. Furthermore, hypothalamic neural stem cells (HtNSCs) were recruited to areas of neuronal damage caused by AT. Through in vivo and in vitro experiments, we clarified the outcomes of AT-induced HtNSC recruitment alongside the loss of mature/intact neurons. Mechanistically, AT induces senescence in these recruited HtNSCs by activating integrated stress response signaling. This consequently hinders the repair of damaged neurons by inhibiting HtNSC proliferation and differentiation. Overall, our findings underscore the pivotal role of the integrated stress response pathway in AT-induced HtNSC senescence and hypothalamic damage. Additionally, the present study offers novel perspectives to understand the mechanisms of AT-induced neurotoxicity and provides preliminary evidence linking AT contamination to the development of NDs.

## Introduction

In recent years, persistent environmental pollution from certain pesticides, owing to high-dose exposure and long-term use, has raised great public health concerns [1]. Atrazine (AT; Chemical Abstracts Service no. 1912-24-9) is a widespread chemical herbicide, primarily designed for weed control in crop fields, and has been utilized worldwide for 65 years [2]. With an annual usage of 90,000 tons, AT ranks as the second largest herbicide used globally. Despite restrictions in the European Union and the United States, AT continues to be used in various countries such as China, India, and Brazil, due to its economic value and potent weed control capabilities [2,3]. Recent studies in environmental ecology and toxicology have highlighted serious concerns regarding the persistent pollution caused by AT, which enters groundwater and aquatic environments at excessive concentrations [4]. Notably, due to its lengthy half-life, ranging from 41 to 231 d, AT remains detectable in European coastal waters even after regulatory restrictions on its use [2]. This persistence is attributed to ocean currents and the planet’s water cycle, as observed in regions like the Aegean Sea, which is affected by water interchange with the Black Sea and the Marmara Sea, where AT is still in use [5]. Therefore, AT pollution represents a global concern that poses a long-term health risk to the population living in agricultural and water areas. More importantly, understanding its toxicity mechanism is crucial for developing effective mitigation strategies.

The spectrum of the toxicological impact of AT extends across diverse taxa, manifesting in amphibians [6], aquatic animals [7], and mammals [8]. This compound can induce dysfunction in the reproductive system [9], respiratory system [10], digestive system [6], and nervous system [11]. Convincing evidence reveals that exposure to AT can induce irreversible damage to the testes and seminiferous tubules by destroying the blood–testis barrier, thus reducing the motility and quality of sperm [12]. Moreover, the toxicity of AT is also recorded in the gastrointestinal system. For instance, low doses of AT exposure to rats showed necrosis and lipidosis in hepatocytes and portal lymphocytic inflammation [13]. Similarly, in amphibian tadpoles, AT exposure can lead to damage of the intestinal microvilli and epithelial cells, as well as induce imbalances in the intestinal microbiota and metabolic disorders [14]. Recent work has demonstrated that AT induces extensive damage to the central nervous system (CNS), including degeneration of neurons in the cerebellum, brain, and hippocampus and dysfunction of the nigrostriatal system [4]. The mechanisms by which AT causes CNS damage and dysfunction are not yet fully understood; however, current evidence points to several key processes, including the induction of oxidative stress [15], neuronal death [15], impairment of microglial phagocytic function [16], and disruption of major neurotransmitter systems [17]. Furthermore, as a neuroendocrine disruptor, AT has been shown to affect endogenous hormone signals through the hypothalamus–pituitary–gonadal axis, causing reproductive dysfunction [18]. As the beginning of the hypothalamus–pituitary–gonadal axis, the hypothalamus plays a key role in neuroendocrine regulation. It interacts with peripheral tissues and reacts to nutritional and environmental signals to coordinate the aspects of physiological homeostasis [19]. However, previous works have concentrated more on the adverse impact of AT on hypothalamic neuroendocrine function; little is known about the molecular mechanism of AT-induced hypothalamic injury.

Growing evidence suggests that persistent exposure to pesticides is linked to the prevalence of age-related neurodegenerative diseases (NDs) like Alzheimer’s disease and Parkinson’s disease [20]. We previously found that AT exposure evoked renal tubular cell senescence and promoted the progression of renal injury by blocking parkin-mediated mitophagy [21]. Similarly, our previous study also revealed that mice exhibited spatial learning and memory impairments after AT treatment [22]. These aging-like phenotypes suggest that senescence in nerve cells may be aggravated. The hypothalamus, vital for coordinating a variety of fundamental life functions, was recently considered to exert a critical effect on the supervision of the aging speed, and hypothalamic neural stem cells (HtNSCs) mediate this process [23]. In mammals, there is a pool of neural stem cells (NSCs) in the hypothalamus, which plays an important effect in the generation of new neurons (neurogenesis) and damage repair [24]. Recent research has convincingly shown that NSC proliferation and their ability to produce new neurons decline rapidly after senescence, while the incidence of NDs and aging-related diseases increases [19,24]. As described above, NSC senescence may be a potential pathway for AT-induced neurotoxicity and hypothalamic injury. However, knowledge of how AT affects NSCs remains limited.

There is limited knowledge about the hypothalamic neurotoxicity of AT exposure, particularly in relation to its effects on HtNSCs. To address this knowledge gap, we orally administered AT to mice at different doses to investigate its effects on NSCs and neurotoxicity. In parallel, the potential molecular mechanism of AT-induced HtNSC senescence was determined by RNA sequencing (RNA-Seq) and further verified using the C17.2 NSC line and inhibitors of target molecules in vitro. Our findings provide the first evidence of AT-induced HtNSC senescence and reveal the role of the integrated stress response (ISR) signaling pathway in this process. Importantly, the current study is poised to enhance public understanding of the proaging role of AT in mammals.

## Results

### AT exposure caused pathological and neuronal damage in mouse hypothalami

**Fig. 1.**
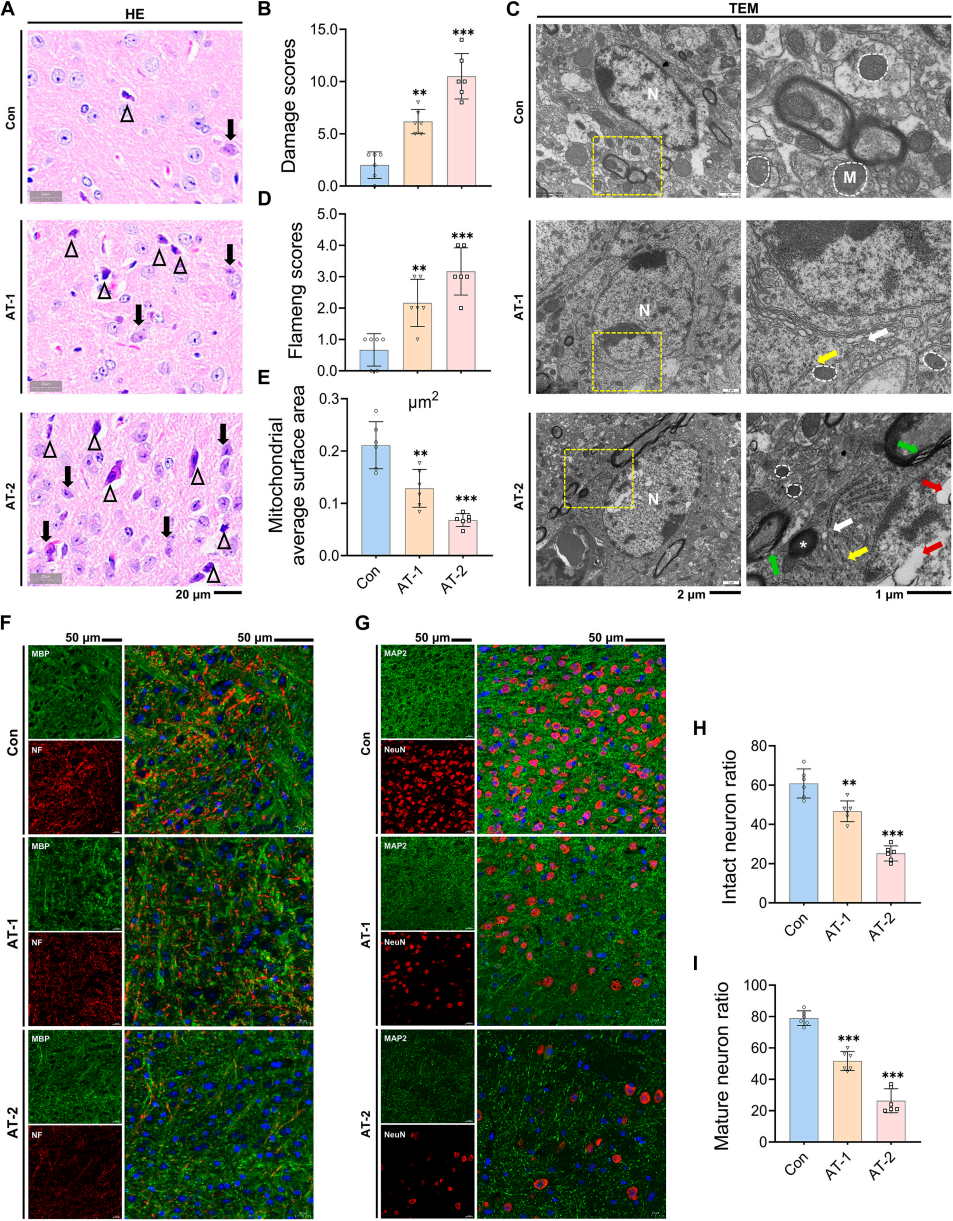
Atrazine exposure caused pathological and neuronal damage in mouse hypothalami. (A) Representative images of hypothalamus tissue sections by hematoxylin and eosin (HE) staining; triangles indicate microglia, and arrows indicate disintegrated nuclear neurons. (B) Histological damage score in the hypothalamus. (C) Representative images of the hypothalamus tissue ultrastructure observed by transmission electron microscopy (TEM). N, nucleus; M, mitochondria; white arrows, endoplasmic reticulum expansion; yellow arrows, polyribosome depolymerization; green arrows, myelin sheath fragmentation; red arrows, nuclear inclusions; asterisk, lipofuscin. (D) Mitochondrial Flameng score in the hypothalamus. (E) Mitochondrial average surface area. (F) Double immunofluorescence staining of hypothalamus tissue samples; representative images of myelin basic protein (MBP) and neurofilament (NF) staining. MBP^+^NF^+^ double-positive cells represent intact neurons. The intact neuron ratio was calculated as MBP^+^NF^+^ cell count/total cell count in a given field of view. (G) Double immunofluorescence staining of hypothalamus tissue samples; representative images of microtubule-associated protein 2 (MAP2) and neuronal nuclei (NeuN) staining. MAP2^+^NeuN^+^ double-positive cells represent mature neurons. The mature neuron ratio was calculated as MAP2^+^NeuN^+^ cell count/total cell count in a given field of view. Scale bars, 50 μm. (H and I) Statistical analysis of the (H) intact neuron ratio and (I) mature neuron ratio. The cell-structure-specific markers used include MBP (myelin sheath), NF (axons), MAP2 (mature neurons), and NeuN (mature neurons). The data are presented as mean ± SD. Statistical analysis was performed using one-way analysis of variance (ANOVA) for multiple group comparisons followed by Tukey’s post hoc pairwise comparison. ^**^P < 0.01, and ^***^P < 0.001 vs. the control (Con) group.

The chemical structure of AT is shown in Fig. S1. To evaluate the effect of AT exposure on morphological and ultrastructural damage to the hypothalamus in mice, hematoxylin and eosin (HE) staining and transmission electron microscopy (TEM) were performed. HE staining results showed that the control (Con) group exhibited only minor disintegrated nuclear neurons and a few microglia in the hypothalamus; most neurons retained normal morphology, with intact nuclei, clear contours, uniformly stained cytoplasm, and no evident signs of degeneration or necrosis (Fig. 1A). In contrast, after AT exposure, nuclear disintegration increased and microglia were substantially activated in a dose-dependent manner. Approximately 1.5- to 2-fold higher levels of nuclear disintegration and microglial activation were observed in the AT-2 group (200 mg/kg AT exposure) compared to the AT-1 group (50 mg/kg AT exposure). These findings were also corroborated by the histological damage score, which showed a notable increase after AT exposure (Fig. 1B). The TEM results revealed that the hypothalami in the AT-2 treatment group showed severe ultrastructural damage (Fig. 1C), manifested by endoplasmic reticulum (ER) expansion (white arrows), polyribosome depolymerization (yellow arrows), myelin sheath fragmentation (green arrows), and nuclear inclusions (red arrows). The Flameng score is a semiquantitative method for evaluating the severity of mitochondrial damage, with a positive correlation between the score and the extent of mitochondrial injury [25]. The results of the mitochondrial Flameng score (Fig. 1D) and average surface area (Fig. 1E) indicated that AT exposure impaired the mitochondrial structure in a dose-dependent manner. Surprisingly, senescence-related lipofuscin (asterisk) was observed in the AT-2 group.

As a neuroendocrine center, the hypothalamus has been shown to exhibit dysfunction after AT exposure [26]. Ultrastructural analysis by TEM revealed that AT caused myelin sheath breakage in hypothalamic neurons, suggesting that neuronal damage may be the driving factor of its dysfunction. We labeled axons (neurofilament, NF^+^) and myelin sheaths (myelin basic protein, MBP^+^) using multiplex immunofluorescence (IF) staining to evaluate the effect of AT exposure on neuronal structural integrity (Fig. 1F). The results showed that AT dose-dependently reduced the ratio of intact neurons in the hypothalami (Fig. 1H). Similarly, the ratio of mature neurons was also significantly decreased by AT exposure (Fig. 1I), as evidenced by the colocalization of neuronal nuclei (NeuN) and microtubule-associated protein 2 (MAP2; Fig. 1G). These results indicated that AT exposure induced hypothalamic neuron damage and impaired recovery from the loss of mature neurons, which may be related to cell senescence.

### Microglia and HtNSCs were recruited in the area of neural injury after AT exposure

**Fig. 2.**
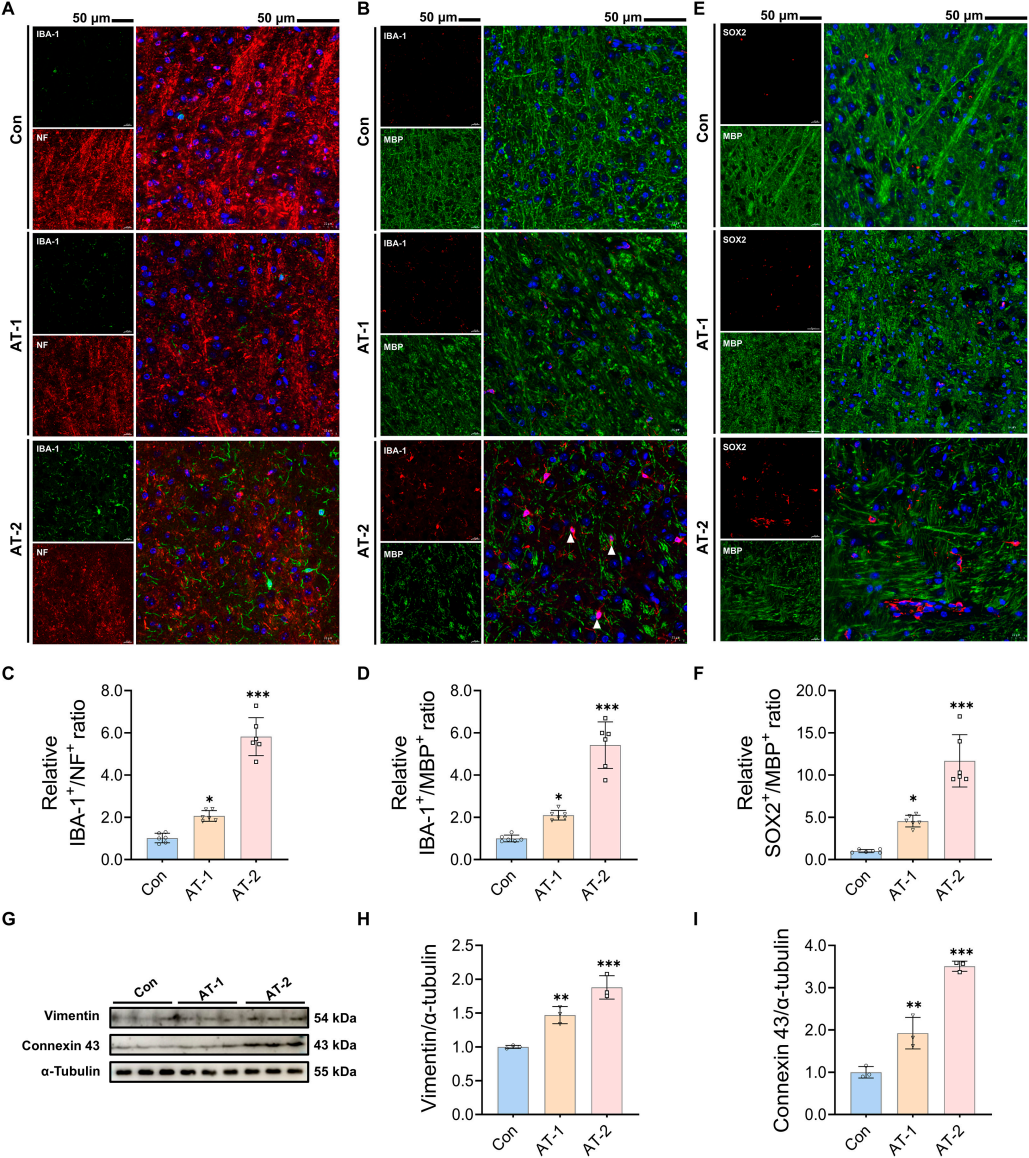
Microglia and hypothalamic neural stem cells (HtNSCs) were recruited to the damaged neural areas after atrazine exposure. (A and B) Double immunofluorescence staining of hypothalamus tissue samples. Scale bars, 50 μm. (A) Representative images of ionized calcium-binding adapter molecule 1 (IBA-1) and NF staining. (B) Representative images of IBA-1 and MBP staining. The cell-structure-specific markers used include MBP (myelin sheath) and NF (axons). IBA-1^+^, microglia. (C) Relative IBA-1^+^/NF^+^ ratio. (D) Relative IBA-1^+^/MBP^+^ ratio. The IBA-1^+^/NF^+^ or IBA-1^+^/MBP^+^ ratio represents the proportion of microglia to structurally intact neurons in a given field of view. (E) Double immunofluorescence staining of hypothalamus tissue samples; representative images of SRY (sex determining region Y)-box 2 (SOX2) and MBP staining. SOX2^+^, neural stem cell. (F) Relative SOX2^+^/MBP^+^ ratio. The SOX2^+^/NF^+^ ratio represents the proportion of HtNSCs to structurally intact neurons in a given field of view. (G) Western blotting measurements of the protein levels of vimentin and connexin 43 in hypothalamus. (H and I) Statistical analysis of (H) vimentin and (I) connexin 43 protein levels. The data are presented as mean ± SD. Statistical analysis was performed using one-way ANOVA for multiple group comparisons followed by Tukey’s post hoc pairwise comparison. ^*^P < 0.05, ^**^P < 0.01, and ^***^P < 0.001 vs. the Con group.

Microglia, serving as the primary mediators of innate immune responses in the CNS, are essential in neuroinflammation and secondary injury after nerve damage [27]. Consistent with the HE staining results, the number of microglia increased after AT exposure in a dose-dependent manner (Fig. 2A). Moreover, microglia were observed to phagocytize myelin debris in the AT-2 treatment group (Fig. 2B). Importantly, based on IF colocalization analysis of NF or MBP and ionized calcium-binding adapter molecule 1 (IBA-1, microglia^+^), microglia in the area of hypothalamic injury were markedly activated induced by AT, as reflected by the increased ratio of IBA-1^+^/NF^+^ and IBA-1^+^/MBP^+^ cells (Fig. 2C and D). On the other hand, recent evidence suggests that a third pool of NSCs exists in the mouse hypothalamus and is engaged in damage repair, systemic aging, and reproduction [24]. As shown in Fig. 2E, IF costaining of the active HtNSC marker (SRY [sex determining region Y]-box 2 [SOX2^+^]) and myelin sheath marker (MBP^+^) revealed that the expression levels of SOX2^+^ and MBP^+^ exhibited a conspicuous negative correlation after AT exposure. Furthermore, SOX2^+^ HtNSCs were significantly recruited in a dose-dependent manner in the AT-treated group. Importantly, these recruited HtNSCs (SOX2^+^) were predominantly located in damaged areas lacking MBP^+^ expression (Fig. 2F). Furthermore, tanycytes, a specialized type of HtNSC, play a crucial role in injury restoration [28]. As markers of tanycytes, vimentin and connexin 43 exhibited significantly increased expression levels in the hypothalamus of the AT-1 and AT-2 treatment groups (Fig. 2G to I). This further indicated that these HtNSCs may be recruited from tanycytes with the characteristics of damage repair. These results suggested that after AT exposure, microglia and HtNSCs were recruited to areas of neuronal damage in the hypothalamus, and this recruitment appears to facilitate the repair process.

### AT exposure restrained the proliferation and differentiation of recruited HtNSCs

**Fig. 3.**
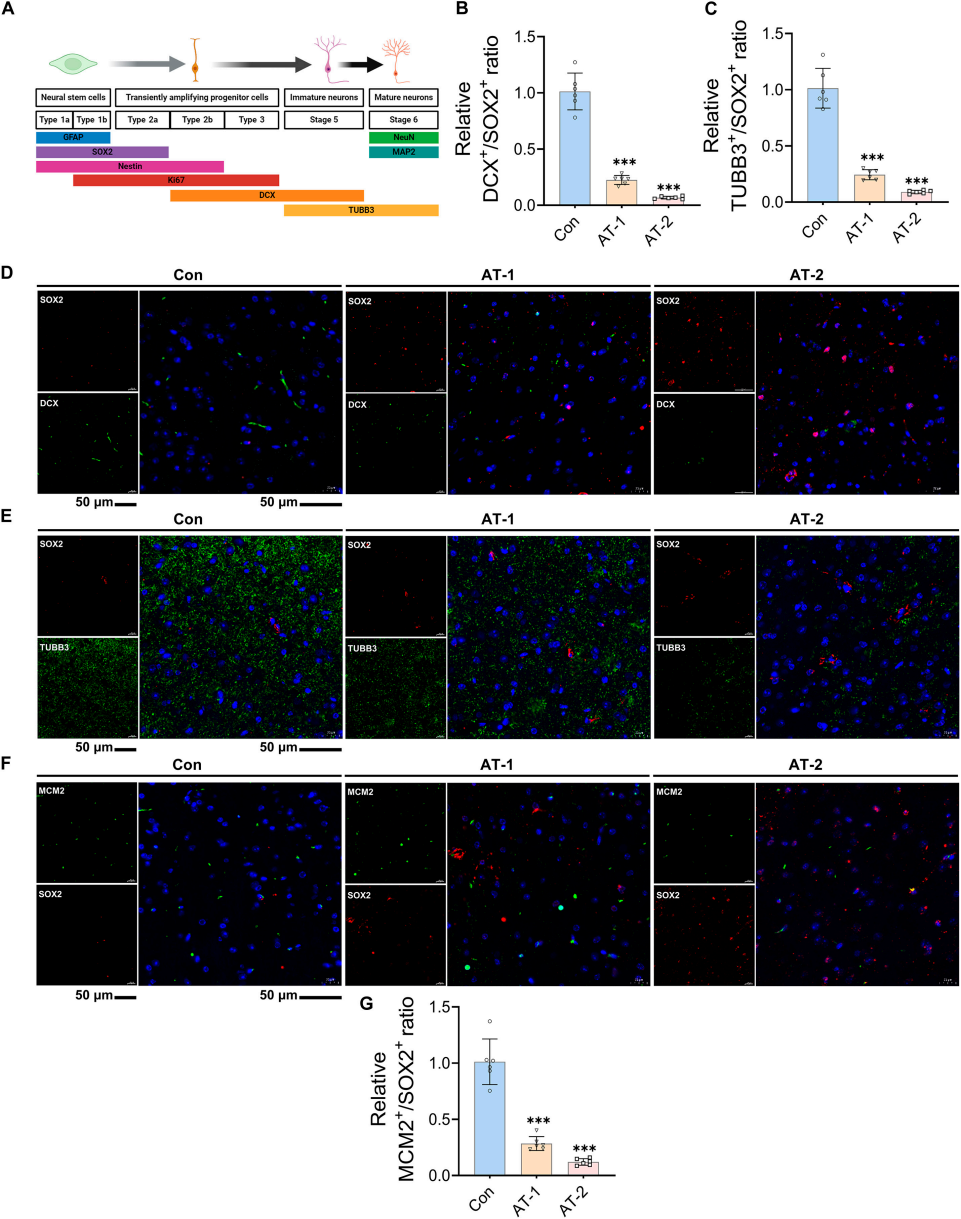
Atrazine exposure restrained the proliferation and differentiation of the recruited hypothalamic neural stem cells (NSCs). (A) Schematic diagram showing stages of adult NSC neurogenesis and cell-lineage-specific markers. (B) Relative doublecortin (DCX)^+^/SOX2^+^ ratio. (C) Relative β3-tubulin (TUBB3)^+^/SOX2^+^ ratio. The DCX^+^/SOX2^+^ or TUBB3^+^/SOX2^+^ ratio represents the proportion of differentiated HtNSCs to total HtNSCs in a given field of view. (D to F) Double immunofluorescence staining of hypothalamus tissue samples. (D) Representative images of SOX2 and DCX staining. (E) Representative images of SOX2 and TUBB3 staining. (F) Representative images of SOX2 and mini chromosome maintenance 2 (MCM2) staining. MCM2 is a proliferation-specific marker. (G) Relative MCM2^+^/SOX2^+^ ratio. The MCM2^+^/SOX2^+^ ratio represents the proportion of actively proliferating HtNSCs to total HtNSCs in a given field of view. Scale bars, 50 μm. The data are presented as mean ± SD. Statistical analysis was performed using one-way ANOVA for multiple group comparisons followed by Tukey’s post hoc pairwise comparison. ^***^P < 0.001 vs. the Con group. GFAP, glial fibrillary acidic protein.

Here are seemingly contradictory results: mature neurons in the hypothalamus were significantly reduced, whereas massive HtNSCs were recruited. We hypothesized that the differentiation and proliferation of these recruited HtNSCs may be restrained. According to the NSC differentiation marker protein profile (Fig. 3A), we detected the expression levels of doublecortin (DCX) and β3-tubulin (TUBB3). The results revealed that DCX and TUBB3 were rarely expressed in areas recruited by SOX2^+^ NSCs in a dose-dependent manner (Fig. 3B to E), suggesting that these recruited HtNSCs were not directly differentiated into neurons under AT exposure. Additionally, the expression levels of the proliferation-specific marker mini chromosome maintenance 2 (MCM2) were also markedly decreased by AT (Fig. 3F and G). Therefore, these results further support our hypothesis.

### AT exposure induced DNA damage and senescence of HtNSCs by activating the ISR pathway

**Fig. 4.**
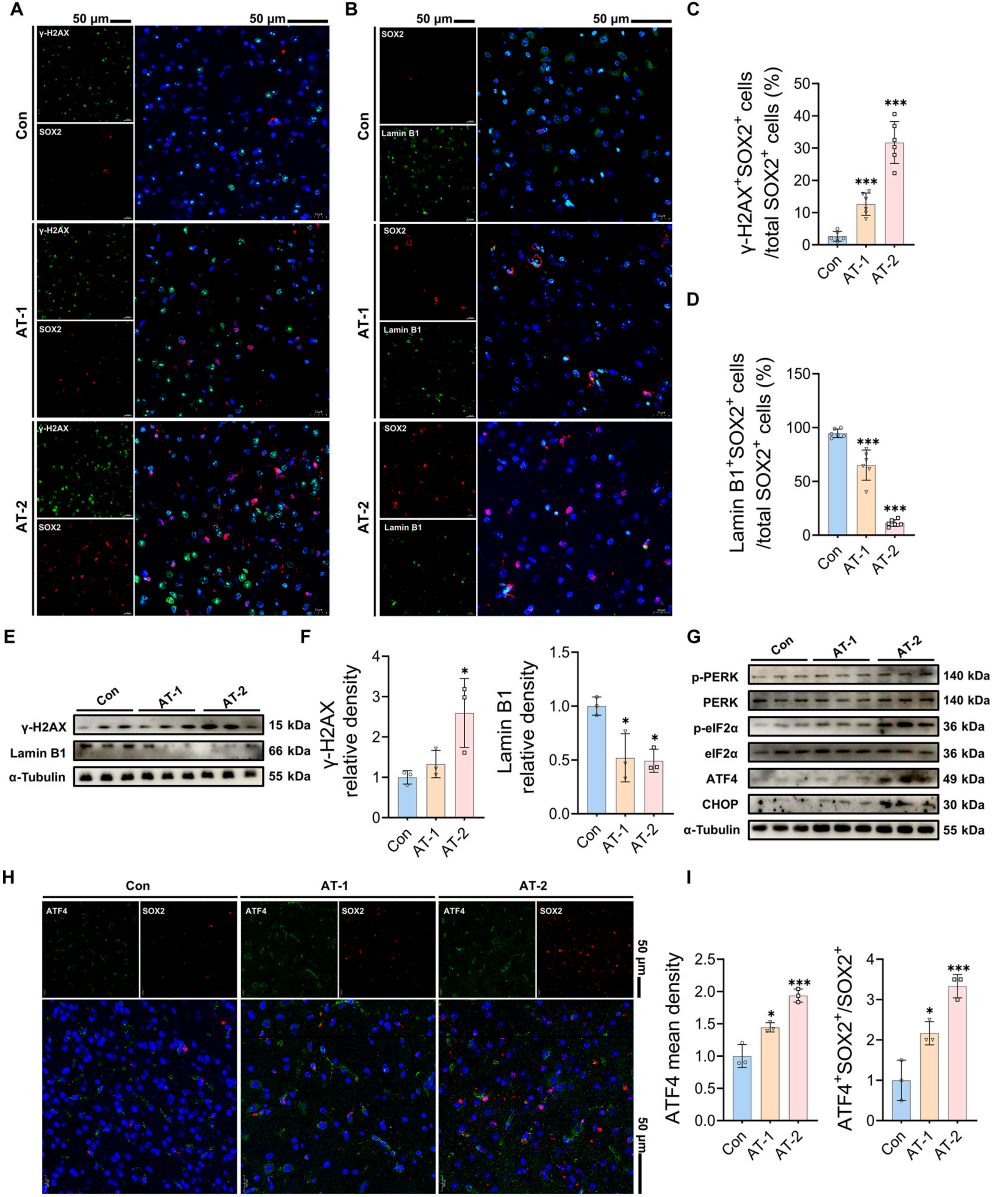
Atrazine exposure induced DNA damage and senescence of recruited HtNSCs by activating integrated stress response (ISR) signaling. (A and B) Double immunofluorescence staining of hypothalamus tissue samples. (A) Representative images of SOX2 and gamma-histone H2A variant X (γ-H2AX) staining; γ-H2AX is a specific marker of DNA damage. (B) Representative images of SOX2 and lamin B1 staining. Lamin B1 is a specific marker of cell senescence. (C and D) Statistical analysis of the percentage of (C) γ-H2AX^+^SOX2^+^ cells and (D) lamin B1^+^SOX2^+^ cells relative to the total SOX2^+^ cells. Scale bars, 50 μm. (E) Western blotting measurements of the protein levels of γ-H2AX and lamin B1 in hypothalamus. (F) Statistical analysis of γ-H2AX and lamin B1 protein levels. (G) Western blotting measurements of the protein levels of the ISR signaling pathway in the hypothalamus. (H) Representative images of SOX2 and activating transcription factor 4 (ATF4) staining. (I) Statistical analysis of ATF4 mean density and ATF4^+^SOX2^+^/SOX2^+^. The data are presented as mean ± SD. Statistical analysis was performed using one-way ANOVA for multiple group comparisons followed by Tukey’s post hoc pairwise comparison. ^*^P < 0.05 and ^***^P < 0.001 vs. the Con group. PERK, protein kinase R-like endoplasmic reticulum kinase; p-PERK, phosphorylated protein kinase R-like endoplasmic reticulum kinase; ATF4, activating transcription factor 4; CHOP, C/EBP homologous protein.

Diminished proliferation and differentiation abilities are clear signs of senescent NSCs [29], suggesting that AT-induced dysfunction of HtNSCs may be associated with senescence. We subsequently determined the levels of the DNA damage marker gamma-histone H2A variant X (γ-H2AX) and the senescence marker lamin B1 in the recruited HtNSCs. As shown in Fig. 4A to D, γ-H2AX was highly expressed, whereas lamin B1 expression was down-regulated in SOX2^+^ HtNSCs after AT exposure. Similarly, the protein levels of γ-H2AX in the hypothalamus of the AT-2 group were strikingly elevated, whereas the lamin B1 levels were decreased (Fig. 4E and F). These results indicated that the recruited HtNSCs were in a state of senescence and DNA damage due to AT exposure.

To explore the specific mechanism of AT-induced HtNSC senescence, RNA-Seq analysis was performed in this study. Given the observed dose-dependent effect of AT neurotoxicity, the 200 mg/kg AT exposure likely more effectively reveals the molecular mechanisms underlying AT-induced HtNSC senescence. Therefore, we selected the 200 mg/kg dose for RNA-Seq to identify potential key pathways and gene expression changes involved in this process. Results indicated a remarkable difference between the Con and AT treatment groups, as observed by principal component analysis and hierarchical clustering (Fig. S2A and B). This indicated that AT exposure changed the transcriptional profile of the hypothalamus. A total of 520 differentially expressed genes were identified between the 2 groups (Fig. S2C). Among them, 205 genes were up-regulated after AT treatment, including the ISR pathway-related genes *DNA damage-inducible transcript 3/C/EBP homologous protein* (*DDIT3/CHOP*), *activating transcription factor 4* (*ATF4*), *Eif2ak3/protein kinase R-like endoplasmic reticulum kinase* (*PERK*), and *eukaryotic initiation factor 2 alpha kinase 4* (*Eif2ak4*), as well as the senescence-related gene *cyclin-dependent kinase inhibitor 1A* (*CDKN1A*) and the DNA damage-related gene histone H2A variant X (*H2AX*, Fig. S2C). Moreover, the pathway of ISR signaling was up-regulated in the AT-treated mouse hypothalami based on the Gene Set Enrichment Analysis (GSEA) results (Fig. S2D). The ISR pathway is a cellular signaling network induced by extracellular stress (Fig. S2E). Its overactivation, leading to loss of proteostasis, is associated with various age-related diseases [30]. Our results indicated that AT exposure notably activated ISR signaling in a dose-dependent manner, as reflected by the elevated phosphorylation of PERK and eukaryotic translation initiation factor 2 alpha (eIF2α) and the increased levels of ATF4 and CHOP (Fig. 4G and Fig. S2F to K). More importantly, the expression levels of ATF4 were markedly elevated in SOX2^+^ HtNSCs after AT exposure (Fig. 4H and I). These findings revealed that the ISR signaling pathway exerts a vital role in AT-induced HtNSC senescence.

### AT exposure induced senescence and ISR signaling activation in C17.2 NSCs

**Fig. 5.**
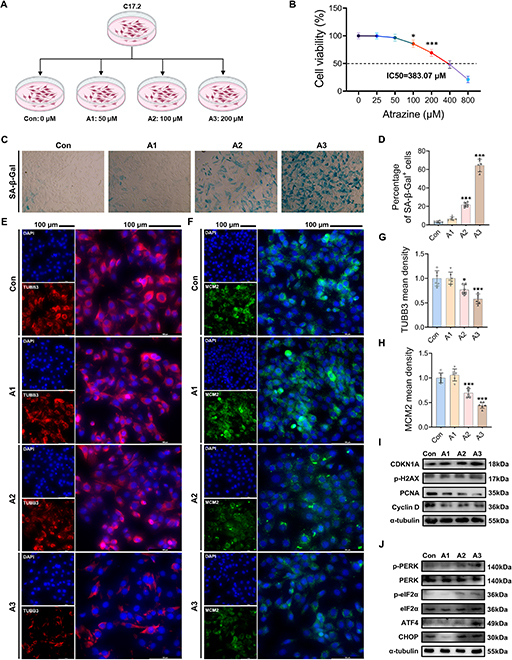
Atrazine exposure induced senescence and ISR signal activation in C17.2 NSCs. (A) C17.2 NSCs were treated with atrazine. (B) Cell viability across various concentrations of atrazine treatment. (C) Representative image of C17.2 NSCs by senescence-associated β-galactosidase (SA-β-Gal) staining. Magnification: ×400. (D) Statistical analysis of percentage of SA-β-Gal^+^ cells. (E) Representative images of 4′,6-diamidino-2-phenylindole (DAPI) and TUBB3 staining. (F) Representative images of DAPI and MCM2 staining; scale bars, 100 μm. (G and H) Statistical analysis of (G) TUBB3 and (H) MCM2 mean density. (I) Western blotting measurements of the protein levels of cyclin-dependent kinase inhibitor 1A (CDKN1A), phosphorylated histone H2A variant Xp-H2AX (p-H2AX), proliferating cell nuclear antigen (PCNA), and cyclin D in C17.2 NSCs. (J) Western blotting measurements of the protein levels of the ISR signaling pathway in C17.2 NSCs. The data are presented as mean ± SD. Statistical analysis was performed using one-way ANOVA for multiple group comparisons followed by Tukey’s post hoc pairwise comparison. ^*^P < 0.05 and ^***^P < 0.001 vs. the Con group.

The C17.2 NSC line was subsequently used to investigate the mechanism of AT-induced HtNSC senescence in vitro (Fig. 5A). Based on the cell viability assay (Fig. 5B), we selected AT concentrations of 50, 100, and 200 μM for subsequent experiments (IC_50_ = 383.07). Consistent with in vivo results, AT caused senescence of C17.2 NSCs, which was manifested by an increase in the proportion of the senescence-associated β-galactosidase^+^ (SA-β-Gal^+^) cells (Fig. 5C and D). Moreover, the expression levels of the differentiation marker TUBB3 and the proliferation marker MCM2 in C17.2 NSCs were remarkably decreased dose-dependently after AT exposure (Fig. 5E to H). Furthermore, genes related to the senescence-associated secretory phenotype (SASP), including *matrix metalloproteinase-3* (*MMP3*), *cyclin-dependent kinase inhibitor 2A* (*CDKN2A*), *interleukin 6* (*IL-6*), *interleukin 8* (*IL-8*), and *C-X-C motif chemokine ligand 1* (*CXCL1*), were also notably elevated in C17.2 NSCs (Fig. S3A to E). Likewise, the expression levels of the senescence-related proteins CDKN1A and phosphorylated histone H2A variant Xp-H2AX (p-H2AX; Fig. 5I and Fig. S3F to G) were significantly promoted with higher doses of AT, while the proliferation-related proteins proliferating cell nuclear antigen and cyclin D (Fig. 5I and Fig. S3H and I) were decreased conversely. Of note, consistent with the animal experimental results, ISR signaling activity was notably elevated in C17.2 NSCs after AT exposure in a dose-dependent manner (Fig. 5J and Fig. S3J to O). These results further confirmed that AT can inhibit the repair of hypothalamic injury by inducing HtNSC senescence.

### Inhibition of ISR signaling alleviated AT-induced senescence in C17.2 NSCs

**Fig. 6.**
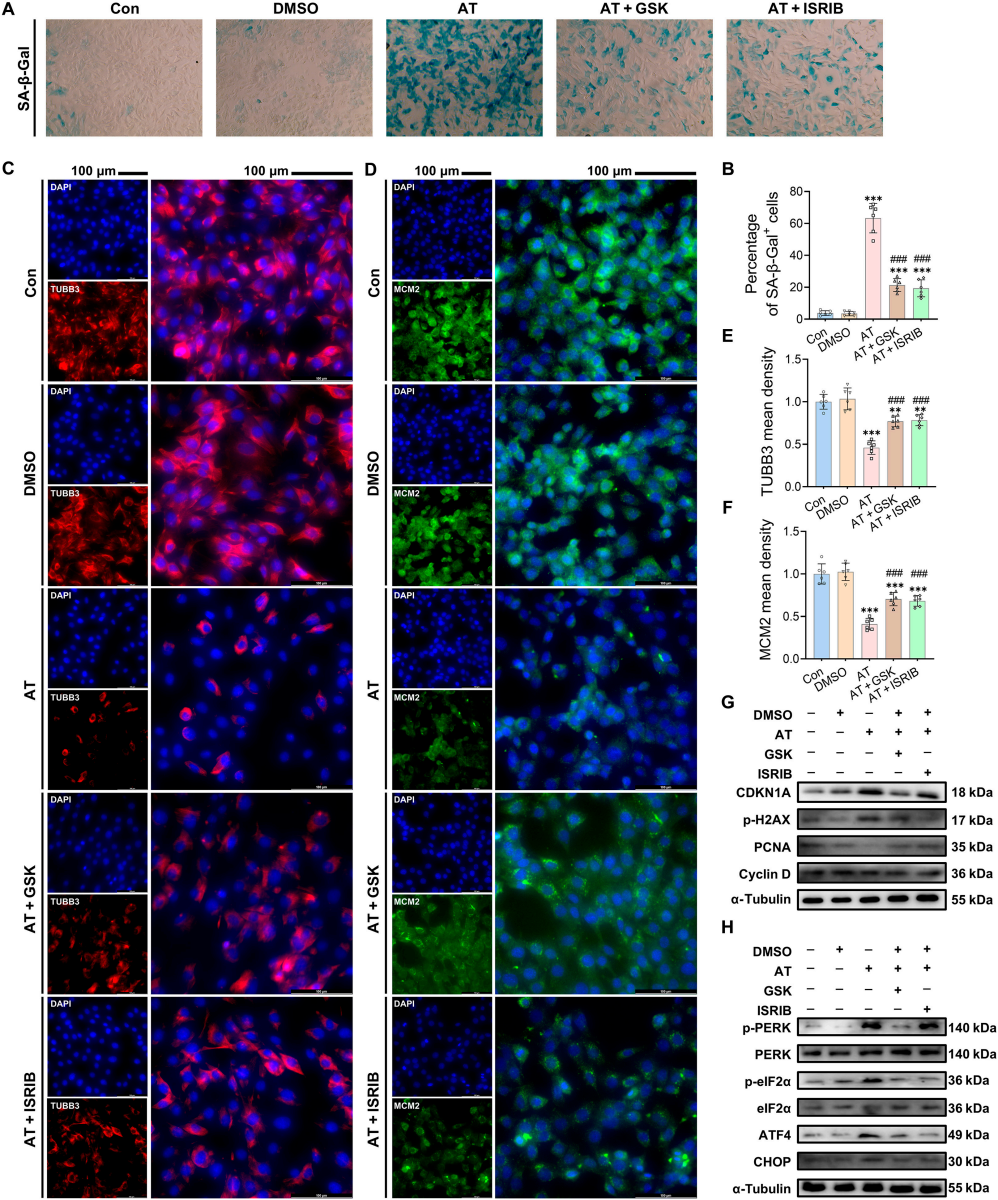
Inhibition of ISR signaling alleviated atrazine (AT)-induced senescence in C17.2 NSCs. (A) Representative images of C17.2 NSCs by SA-β-Gal staining after GSK2606414 (GSK) and ISRIB pretreatment. Magnification: ×400. (B) Statistical analysis of the percentage of SA-β-Gal^+^ cells. (C) Representative images of DAPI and TUBB3 staining after GSK and ISRIB pretreatment. (D) Representative images of DAPI and MCM2 staining after GSK and ISRIB pretreatment; scale bars, 100 μm. (E and F) Statistical analysis of (E) TUBB3 and (F) MCM2 mean densities. (G) Western blotting measurements of the protein levels of CDKN1A, p-H2AX, PCNA, and cyclin D in C17.2 NSCs after GSK and ISRIB pretreatment. (H) Western blotting measurements of the protein levels of the ISR signaling pathway in C17.2 NSCs after GSK and ISRIB pretreatment. The data are presented as mean ± SD. Statistical analysis was performed using one-way ANOVA for multiple group comparisons followed by Tukey’s post hoc pairwise comparison. ^**^P < 0.01 and ^***^P < 0.001 vs. the Con group. ^###^*P* < 0.001 vs. the AT group. DMSO, dimethyl sulfoxide.

To verify the key role of ISR signaling in AT-induced NSC senescence, we used the PERK inhibitor GSK2606414 (GSK) and the eIF2α inhibitor ISRIB to block the ISR signaling pathway (Fig. S4A). SA-β-Gal staining showed that pretreatment with GSK or ISRIB significantly mitigated AT-induced senescence of C17.2 NSCs (Fig. 6A and B). Meanwhile, the impaired differentiation and proliferation of C17.2 NSCs induced by AT exposure were alleviated by GSK and ISRIB (Fig. 6C to F). Furthermore, treatment of C17.2 cells with GSK or ISRIB prevented the AT-induced up-regulation of SASP-related genes (Fig. S4B to F) and senescence-related proteins (Fig. 6G and Fig. S4G and H). Additionally, this treatment reversed the down-regulation of proliferation-related proteins (Fig. 6G and Fig. S4I and J). Finally, the Western blot analysis results indicated that GSK and ISRIB pretreatment effectively blocked the AT-induced activation of the ISR signaling pathway (Fig. 6H and Fig. S4K to P). Overall, these data confirmed that the ISR signaling pathway was notably activated in HtNSCs and played a vital role in the senescence of HtNSCs caused by AT exposure.

## Discussion

Emerging evidence suggests that environmental factors, especially persistent pesticide pollution, are linked to the development of NDs such as Parkinson’s disease [20]. Dementia, a common consequence of NDs, will affect 150 million individuals within the next 30 years, causing both physical suffering and economic burden [31]. Research has demonstrated that stem cell therapy holds great potential for the treatment of degenerative conditions such as motor neuron disease and Parkinson’s disease [32]. Hence, investigating the toxic mechanisms of widely used pesticides on NSCs is vital. AT, the second most commonly used herbicide globally, poses a serious threat to human health through exposure via the food chain or drinking water [2]. In this study, we demonstrate, for the first time, that AT causes neuronal injury and microglia activation and recruits HtNSCs to areas of neuronal injury in hypothalami. Intriguingly, AT induces senescence in these recruited HtNSCs by activating the ISR signaling pathway. This senescence of HtNSCs further hinders neuronal damage repair by reducing their proliferation and differentiation. Importantly, our findings provide preliminary evidence of a potential association between AT contamination and the development of aging-related NDs, which may help raise public health awareness regarding AT contamination.

The hypothalamus is a crucial neuroendocrine center that regulates reproduction, emotion, body temperature, exercise, and aging. While numerous studies have documented the pathological damage effects of AT exposure to the liver, kidney, spleen, brain, cerebellum, and hippocampus [2,4,33,34], research on the hypothalamus has predominantly focused on AT-induced hormonal disruptions, with limited attention to its morphological and structural damage [17]. Mitochondria and the ER play a vital role in maintaining protein homeostasis [35]. Studies have shown that a decrease in protein clearance capacity and loss of protein homeostasis are indicative of cell senescence [36]. In this study, we observed severe morphological and ultrastructural damage in the hypothalamus due to AT exposure, including nuclear disintegration, neuronal myelin fragmentation, and structural damage to the ER and mitochondria. Furthermore, increased lipofuscin and a decrease in mitochondrial surface area suggest that AT-induced hypothalamic damage may be related to cell aging. Lipofuscin is often called senescence pigment and considered a hallmark of aging [37]. To the best of our knowledge, there are currently no reports of lipofuscin accumulation in neural tissue due to AT exposure. Experimental studies have shown that some persistent environmental pollutants, including pesticide fungicides (triadimenol) [38] and polystyrene nanoplastics [39], accelerate aging in the intestinal and brain tissues of *Caenorhabditis elegans* and zebra fish models. The accumulation of the aging biomarker lipofuscin observed in these models is consistent with our findings. Additionally, neuroinflammation in microglia is considered a critical marker of nerve damage and the development of NDs [40,41]. A previous study showed that AT exposure induced neurotoxicity and neuroinflammation by stimulating microglia and suppressing the nuclear factor erythroid 2-related factor 2 signaling pathway [40]. Consistent with this study, we also found that AT induced marked activation of microglia in the areas of neuronal injury in the hypothalamus.

A complete neuron structure and an adequate number of mature neurons are crucial for sustaining the normal function of the CNS. Through IF costaining, we found that the proportion of intact neurons and mature neurons was significantly decreased after AT exposure, as reflected by the reduced density of MBP^+^, NF^+^, MAP2^+^, and NeuN^+^. These results directly proved the neurotoxicity of AT. Learning and memory deficits, substantial symptoms of CNS injury, are associated with neuronal structural damage and microglial dysfunction. Our previous study showed that, in the Morris water maze test, AT exposure at 50 and 200 mg/kg caused mice to spend more time searching for the platform, and the number of times the mice crossed the platform area after its removal was lower than that of the control group [22]. Additionally, multiple studies have found that AT exposure induced learning and memory impairments in rates [42], a phenotype that directly supports the reduced intact neurons and mature neurons observed in this study. NSCs, with self-renewal and differentiation potential, play a pivotal role in neurogenesis and neural repair in NDs [43]. Hence, this raises an interesting biological question: does AT-induced neuronal injury trigger NSC recruitment to produce new neurons? Surprisingly, our results indicated that HtNSCs were indeed recruited to the areas of neuronal injury after AT exposure, as evidenced by the main localization of recruited SOX2^+^ NSCs in damaged areas lacking MBP expression. Tanycyte NSCs in the mouse hypothalamus possess strong regenerative potential and are involved in the repair process following neural injury [28,29]. A previous study indicated that mechanical injury induced by inserting an acupuncture needle into the median eminence led to strong activation of tanycyte NSCs, in contrast to their quiescent state observed in sham control mice [28]. Therefore, in our study, the recruitment of tanycyte NSCs provides further evidence that the mouse nervous system attempts to restore AT-induced hypothalamic neuronal damage.

However, the results of this study showed that AT-induced neurotoxicity was not alleviated by the recruited HtNSCs. As a recently discovered type of NSC, HtNSCs have the potential to attenuate or even revert multiple aspects of systemic senescence [19]. An early study revealed that lipid accumulation can inhibit HtNSC differentiation and proliferation by inducing senescence, which subsequently leads to damage and dysfunction of hypothalamic neurons [29]. This suggests that the observed loss of intact neurons and the recruitment of HtNSCs may be related to AT-induced HtNSC senescence. As expected, AT exposure impaired the proliferation and differentiation of HtNSCs in a dose-dependent manner. In parallel, AT exposure induced DNA damage and senescence in these recruited HtNSCs, as manifested by the up-regulation of γ-H2AX and down-regulation of lamin B1 in SOX2^+^ HtNSCs. As reported, the capacity of NSCs to proliferate and generate new neurons markedly diminishes after senescence, while the occurrence of neurodegeneration and age-related diseases rises [24]. This is consistent with our findings, where we observed that after AT exposure, particularly at a dose of 200 mg/kg, the proportion of HtNSCs differentiating into neurons in the area of neural injury was significantly reduced. These results explain the observed decrease in the number of intact and mature neurons, as well as the strong correlation between AT contamination and the occurrence and progression of NDs. It can be inferred that when AT exposure induces neuronal damage, HtNSCs are recruited in an attempt to exert a repair function to maintain neurogenesis; however, this process is inhibited upon induction of HtNSC senescence. A previous study indicated that the potential for senescent HtNSCs to differentiate into neurons is reduced, with some differentiating into oligodendrocytes [29]. This provides valuable insight; however, our study did not further explore the ultimate fate of these senescent HtNSCs. Instead, we focused on the specific molecular mechanisms by which AT exposure induces HtNSC senescence. Accumulating evidence suggests that the early characteristics of many NDs include impaired regenerative capacity of NSCs, which contributes to promoting disease progression by inducing brain tissue degeneration and dysfunction [44]. Therefore, it can be confirmed that AT-induced NSC senescence contributes to hypothalamic injury. However, the precise mechanisms by which AT induces HtNSC senescence remain unclear.

Current theories on the mechanisms of aging highlight the progressive disruption of cellular protein homeostasis and the decline in protein quality control as key factors [45]. As a central regulator of protein homeostasis, the ISR signaling network controls protein homeostasis by regulating protein synthesis rates at both the cellular and organismal levels [30]. Mechanistically, extracellular stressors such as toxin exposure induce the buildup of misfolded proteins in the ER, which activates the stress sensor eIF2 kinases like PERK, leading to its phosphorylation. This phosphorylation subsequently stimulates the phosphorylation of the substrate eIF2α and promotes the expression of downstream feedback factors CHOP and ATF4, thereby reducing overall protein synthesis [30,46]. However, dysregulation of ISR signaling is involved in the etiopathogenesis of multiple complicated diseases, such as NDs, cognitive impairment, and cancer [30]. In our study, RNA-Seq and Western blot analysis indicated that the ISR pathway was stimulated by AT exposure in mouse hypothalami. It is worth emphasizing that the ISR pathway was also stimulated in HtNSCs after AT exposure, as confirmed by the elevated expression levels of ATF4 in SOX2^+^ HtNSCs. HtNSCs have been identified as crucial for the hypothalamic regulation of senescence [19]. Furthermore, the activity of ISR signaling increases with age, implying a potential connection to the senescence process. Recent work has indicated that persistent activation of the ISR signaling contributes to long-term memory impairment by reducing cognitive abilities and damaging the structure and function of neurons in aged mice [47]. Similarly, our results demonstrated that hyperactivation of ISR signaling mediates AT-induced HtNSC senescence and hypothalamic injury.

Additionally, these results clarify the seemingly contradictory phenomenon where AT induces the recruitment of HtNSCs for repair, yet these recruited HtNSCs undergo senescence and show inhibited proliferation and differentiation. NSCs in the mammalian brain exist in 2 states: a quiescent state and an active state (or recruitment state, marked by specific SOX2 expression) [48]. Generally, most NSCs maintain a reversible cell cycle arrest or quiescent state. This state is crucial for long-lived proliferative cells, as it protects them from exhaustion of their proliferation potential and allows them to avoid accumulation of damage to DNA, proteins, and mitochondria, which could otherwise lead to malignant transformation or senescence [49]. However, following neural damage, endogenous quiescent NSCs become active and participate in the brain repair process [50]. Thus, one possible explanation for this phenomenon is that AT exposure leads to hypothalamic neuronal damage, which, in turn, induces the activation and recruitment of NSCs to the area of injury. These activated/recruited NSCs, under AT exposure, experience proteostasis disruption through the activation of the ISR signaling pathway, leading to the accumulation of toxic proteins. Ultimately, this results in the senescence of these recruited NSCs and a reduction in their proliferative and differentiation capacities.

C17.2 NSCs are a cell line derived from the developing mouse cerebellum that has multipotency for proliferation and differentiation [51]. In the current study, we employed the C17.2 NSC line to verify the animal experimental results and validate the vital role of ISR signaling in AT-induced HtNSC senescence using inhibitors. Consistent with animal experimental results, AT exposure induced senescence, DNA damage, and a reduction in the multipotency of C17.2 NSC cells in a dose-dependent manner. Moreover, the expression levels of p-PERK, p-eIF2α, ATF4, and CHOP in the ISR signaling pathway were notably increased in a concentration-dependent manner of AT exposure. Of note, inhibition of ISR signaling by GSK or ISRIB alleviated the aging-related phenotypes of C17.2 NSC cells induced by AT exposure. These phenotypes included increased SA-β-Gal activity, inhibited proliferation and differentiation, up-regulated SASP-related genes, and DNA damage. In recent years, the toxicity of pesticide exposure to NSCs and the disruption of neurogenesis have received increasing attention. A previous study demonstrated that prenatal exposure of rats to low doses of the fungicides cyprodinil, mepanipyrim, and pyrimethanil affects NSC proliferation through crosstalk between the PI3K/Akt and Wnt/β-catenin pathways, thereby disrupting neurogenesis in the offspring [52]. Additionally, a study on combined pesticide exposure showed that paraquat and maneb affect the expression of cell-cycle-related genes in NSCs and induce reactive oxygen species accumulation, impairing NSC proliferation by altering redox status mechanisms [53]. Although our study also found that AT exposure inhibited HtNSC proliferation and differentiation, these studies differ from ours. For the first time, our results elucidate the neurotoxic mechanisms of pesticide exposure from the perspective of NSC senescence, showing that it impairs the nervous system’s self-repair ability. Furthermore, our in vivo and in vitro experiments demonstrated that the ISR pathway is the target of HtNSC senescence induced by AT exposure.

However, the current study also has certain limitations. First, this study did not conduct a more detailed dose–response analysis or include lower-dose treatment groups. This would be necessary to determine the threshold level required for AT-induced neurotoxicity and its toxic effects at environmentally relevant exposure concentrations. The dosing regimen in this study was based more on the toxicological effects of AT rather than environmental exposure levels. To achieve observable toxicological effects within a shorter timeframe, the doses used were higher than typical environmental exposure levels. This may limit the environmental relevance of these findings and affect their extrapolation to real-world scenarios. Additionally, there is limited evidence directly associating AT with the development of NDs. Our study investigated only the effect of HtNSC senescence in AT exposure-induced hypothalamic toxicity in mice. Therefore, further research is warranted to explore the effects of AT-induced NSC senescence on the overall nervous system. Additionally, research should investigate the relationship between human exposure concentrations of AT and the development of NDs. This includes environmental investigations of AT and examining patients with NDs for indications of AT residual.

In conclusion, the present study provides the first evidence that exposure to AT, a widely used commercial herbicide, causes neurotoxicity and hypothalamic injury by inducing HtNSC senescence via activating the ISR signaling pathway. Blocking the ISR pathway may effectively prevent NSC senescence and could be used as a potential therapeutic target to alleviate AT-induced neurotoxicity. Furthermore, our findings identify a previously unrecognized mechanism of AT-induced hypothalamic toxicity and provide preliminary evidence linking pesticide exposure to the development of NDs.

## Materials and Methods

### Reagents and chemicals

In this study, AT (purity > 99.17%) was purchased from Macklin Inc. (Shanghai, China). Cell Counting Kit-8 (CCK-8) was purchased from DOJINDO (Kumamoto, Japan). A reverse transcriptase kit was obtained from TransGen Biotech (Beijing, China). The radioimmunoprecipitation assay lysis buffer and the senescence β-galactosidase staining kit were purchased from Beyotime Biotechnology (Shanghai, China). Moreover, the PERK inhibitor GSK (S7307; purity: 99.92%) and the eIF2α inhibitor ISRIB (S0706; purity: 98.81%) were purchased from SelleckChem (Shanghai, China). Furthermore, all antibodies used for Western blot and IF staining in this study are presented in Table S1.

### Animal and experimental design

All experimental procedures for lowering pain in the mice were performed following the approval of the Guide for the Care and Use of Laboratory Animals at Northeast Agricultural University, Harbin, China (NEAUEC20200346). All animal experiments adhered to the Animal Research: Reporting of In Vivo Experiments guidelines. All experimental animals were obtained from Changsheng Biotech (Liaoning, China) and acclimated in the laboratory for 1 week (21 to 23 °C, 30% to 60% humidity, and 12 h light/dark cycle). Thirty 28-d-old male C57BL/6 mice were randomly divided into 3 groups (*n* = 10): Con, AT-1 (50 mg/kg AT), and AT-2 (200 mg/kg AT) groups. The mice in the AT-1 and AT-2 treatment groups were treated with AT daily by gavage for 3 weeks. Subsequently, all the animals were sacrificed for sample collection. The doses of AT-1 and AT-2 were 1/35 or 1/8.75 of the LD_50_ value in mice (17.5 g/kg) after intragastric administration, according to our previous study [54]. These dose selections align with findings from the subacute toxicity study of AT, which indicated that doses exceeding 50 mg/kg are pathogenic [55,56]. Furthermore, exposure to AT in the range of 50 to 200 mg/kg has been shown to impair dopaminergic system function and induce neurotoxicity [57].

### Transmission electron microscopy

Hypothalamus tissues were isolated and fixed with glutaraldehyde, as described previously [58]. All the tissues were subsequently postfixed with OsO_4_ and embedded with epoxy resin after dehydration. Finally, the slices of hypothalamus were stained. An HT7650 TEM microscope (Hitachi, Japan) was employed to capture images. The Flameng score was employed to estimate the Flameng mitochondrial injury score, as described previously [25]. Briefly, 6 fields of view (each containing 20 mitochondria) were randomly selected for each tissue slice. A double-blind protocol was subsequently implemented, with experienced pathologists rating the samples according to the reported scoring criteria (range 0 to 4). Additionally, the ImageJ software (National Institutes of Health, Maryland, United States) was used to calculate the average mitochondrial surface area.

### HE staining

As previously described, HE staining was used to examine the morphological damage of the hypothalamus [59]. Briefly, the paraformaldehyde-fixed hypothalamus was embedded with paraffin and cut to a thickness of 4 μm using a Leica automatic slicer (HistoCore AUTOCUT, Leica, Germany). After HE staining, the hypothalamus slices were scanned using an automatic microscopic scanner. Based on a previous study [60], 6 fields of view were randomly selected for each tissue slice, and an experienced pathologist was asked to score the morphological damage in the hypothalamus using a double-blind method. The scoring criteria were as follows: microglia cells (triangle, 1 point) and disintegrated nuclear neurons (arrows, 2 points).

### Immunostaining

The IF staining procedures were consistent with the previous literature [61]. Briefly, paraffin sections of hypothalamus tissue were rinsed with water for 5 min after dewaxing. Then, the slices were treated with citric acid buffer solution (0.01 M, pH 6.0) for antigen retrieval. For permeabilization, the slices were incubated with 0.1% Triton X-100 (10 min). After blocking with 5% goat serum, the slices were incubated with the corresponding primary and secondary antibodies and 4′,6-diamidino-2-phenylindole for immunostaining. For cell IF, cells were fixed with 4% neutral formaldehyde at 4 °C for 30 min) [62]. The protocols for permeabilization, blocking, and immunostaining were the same as those used for IF staining of tissue slices. Finally, for visualization, a Leica fluorescence microscope (DMi8, Leica, Germany) was employed. The ImageJ software was applied to analyze the fluorescence density or the positive cell ratio.

### Western blot analysis

Total protein from hypothalamus tissues and C17.2 NSC cells was extracted using radioimmunoprecipitation assay lysis buffer. The detailed protocols for Western blot were consistent with our previous report [63]. Briefly, protein samples were separated by 8% to 15% sodium dodecyl sulfate–polyacrylamide gel electrophoresis and transferred to nitrocellulose membranes. After blocking with 5% nonfat dry milk at 37 °C for 2 h, the membranes were incubated with the corresponding primary and secondary antibodies for 12 h (4 °C) and 1 h (37 °C), respectively. The immunoblots were subsequently visualized using Amersham Imager 600 (GE, Switzerland) and quantified using the ImageJ software [64].

### RNA-Seq analysis

Hypothalamus tissue samples from the Con group and the 200 mg/kg AT treatment group were selected for RNA-Seq in this study (*n* = 3). Total RNA from hypothalamus tissues was extracted and sequenced by MAGIGENE Biotech. Co., Ltd. (Shenzhen, China). After obtaining the raw reads, the Fastp software was used to eliminate low-quality data. Consistent with a previous study, HISAT2 (v2.0.5) was employed to analyze the data, and DESeq2 R package 1.16.1 was used to analyze the differentially expressed genes (*P* < 0.05, fold change threshold: 1) [65]. Furthermore, enrichment analysis was performed on these expressed data using the GSEA software (V4.1.0). A more detailed protocol for RNA-Seq is shown in the Supplementary Materials.

### Cell culture and treatment

The mouse NSC line C17.2 was obtained from MeisenCTCC (Jinhua, China) and cultured in Dulbecco’s modified Eagle medium/F12 (HyClone, United States) supplemented with 10% fetal bovine serum, 2% equinum serum, 2 mM glutamine, and 1% anti–anti (37 °C and 5% CO_2_). AT was dissolved in phosphate-buffered saline and incubated with the cells for 12 h. Cells were treated with different stimuli, including dimethyl sulfoxide (<0.1%), GSK at 2 μM (IC_50_: 0.4 nM), and ISRIB at 200 nM (IC_50_: 5 nM), according to specific experimental protocols. The doses of these stimuli used in this study were determined according to the cell viability assay described below or the manufacturer’s instructions.

### Cell viability assay

The CCK-8 assay was applied to assess C17.2 cell viability [63]. Briefly, C17.2 cells were seeded in 96-well plates. After 12 h of treatment, cell viability was determined using a CCK-8 assay following the manufacturer’s specifications. The absorbance was detected at 450 nm using a microplate reader (ELx808, Biotek, United States).

### Quantitative real-time polymerase chain reaction

In this study, the primers for quantitative real-time polymerase chain reaction (PCR) were designed using the Oligo6 software (Table S2). Primer specificity was verified using Primer-BLAST (https://www.ncbi.nlm.nih.gov/tools/primer-blast). The details of total RNA extraction and reverse transcription are described in a previous study [66]. The quantitative real-time PCR was performed using a QS-5 Flex PCR instrument (Applied Biosystems, United States). Relative messenger RNA expression was determined using the 2^−ΔΔCT^ method [67].

### Senescence β-galactosidase staining

SA-β-Gal activity is a marker of cell senescence. C17.2 cells were seeded in a 6-well plate. They were subsequently subjected to different treatments based on the predetermined design. After specific experimental protocols, C17.2 NSCs were fixed with β-galactosidase staining fixative for 15 min according to the manufacturer’s instructions. Then, the cells were washed with phosphate-buffered saline 3 times (3 min each time), and 1 ml of working staining solution was added to each well to incubate for 12 h at 37 °C. Finally, the staining images were recorded with an EVOS XL Core imaging system (Thermo Fisher Scientific, United States).

### Statistical analysis

All statistical data were analyzed and visualized using GraphPad Prism 9.0 (GraphPad Software, San Diego, California, United States) or SPSS 17.0 software (SPSS, Inc., Chicago, Illinois). Statistical analysis was conducted using one-way analysis of variance (ANOVA) followed by Tukey’s post hoc pairwise comparison. All data in the current study are provided as mean ± standard deviation (SD). The detailed specifications of the statistical means are shown in the figure legends. Differences were considered significant at *P* < 0.05.

## Data Availability

All data supporting the findings of this study are available within the article and its supplementary materials.

## References

[1] Xu L, Xu X, Wu X, Kuang H, Xu C. Sex-dependent environmental health risk analysis of flupyradifurone. Environ Sci Technol. 2022;56(3):1841–1853. 35041393 10.1021/acs.est.1c07726

[2] de Albuquerque FP, de Oliveira JL, Moschini-Carlos V, Fraceto LF. An overview of the potential impacts of atrazine in aquatic environments: Perspectives for tailored solutions based on nanotechnology. Sci Total Environ. 2020;700: Article 134868. 31706089 10.1016/j.scitotenv.2019.134868

[3] Wang K, Cai M, Sun J, Chen H, Lin Z, Wang Z, Niu Q, Ji T. Atrazine exposure can dysregulate the immune system and increase the susceptibility against pathogens in honeybees in a dose-dependent manner. J Hazard Mater. 2023;452: Article 131179. 36948121 10.1016/j.jhazmat.2023.131179

[4] Das S, Sakr H, Al-Huseini I, Jetti R, Ai-Qasmi S, Sugavasi R, Sirasanagandla SR. Atrazine toxicity: The possible role of natural products for effective treatment. Plan Theory. 2023;12(12): Article 2278. 10.3390/plants12122278 PMC10301673 37375903

[5] Nodler K, Licha T, Voutsa D. Twenty years later—Atrazine concentrations in selected coastal waters of the Mediterranean and the Baltic Sea. Mar Pollut Bull. 2013;70(1–2):112–118. 23481690 10.1016/j.marpolbul.2013.02.018

[6] Zhao Q, Huang M, Yin J, Wan Y, Liu Y, Duan R, Luo Y, Xu X, Cao X, Yi M. Atrazine exposure and recovery alter the intestinal structure, bacterial composition and intestinal metabolites of male *Pelophylax nigromaculatus*. Sci Total Environ. 2022;818: Article 151701. 34798088 10.1016/j.scitotenv.2021.151701

[7] Gao M, Yang N, Lei Y, Zhang W, Liu H, Lin H. Tannic acid antagonizes atrazine exposure-induced autophagy and DNA damage crosstalk in grass carp hepatocytes via NO/iNOS/NF-κB signaling pathway to maintain stable immune function. Fish Shellfish Immunol. 2022;131:1075–1084. 36396070 10.1016/j.fsi.2022.11.024

[8] Dai XY, Lin J, Zhu SY, Guo JY, Cui JG, Li JL. Atrazine-induced oxidative damage via modulating xenobiotic-sensing nuclear receptors and cytochrome P450 systems in cerebrum and antagonism of lycopene. Food Chem Toxicol. 2022;170: Article 113462. 36216167 10.1016/j.fct.2022.113462

[9] Fang C, Li Y, He G, Gan RY, Luo F, Lei L, Hou X, Ye Y. Silk fibroin microneedles loaded with epigallocatechin gallate mitigate atrazine-induced testicular toxicity. J Hazard Mater. 2024;480: Article 136252. 39461294 10.1016/j.jhazmat.2024.136252

[10] Remigio RV, Andreotti G, Sandler DP, Erickson PA, Koutros S, Albert PS, Hurwitz LM, Parks CG, Lubin JH, Hofmann JN, et al. An updated evaluation of atrazine-cancer incidence associations among pesticide applicators in the Agricultural Health Study cohort. Environ Health Perspect. 2024;132(2): Article 27010. 38381478 10.1289/EHP13684 PMC10880817

[11] Lin J, Zhao HS, Qin L, Li XN, Zhang C, Xia J, Li JL. Atrazine triggers mitochondrial dysfunction and oxidative stress in quail (*Coturnix C. coturnix*) cerebrum via activating xenobiotic-sensing nuclear receptors and modulating cytochrome P450 systems. J Agric Food Chem. 2018;66(25):6402–6413. 29865786 10.1021/acs.jafc.8b01413

[12] Harper AP, Finger BJ, Green MP. Chronic atrazine exposure beginning prenatally impacts liver function and sperm concentration with multi-generational consequences in mice. Front Endocrinol. 2020;11: Article 580124. 10.3389/fendo.2020.580124 PMC7726345 33324343

[13] Riera J, Matus E, Matus L, Molino J. Toxicity of commercial atrazine in rattus novergicus organs as a function of concentration: Histopathological, ultrastructural and hematological evaluation. An Acad Bras Cienc. 2022;94(2): Article e20201125. 35319620 10.1590/0001-3765202220201125

[14] Huang MY, Zhao Q, Duan RY, Liu Y, Wan YY. The effect of atrazine on intestinal histology, microbial community and short chain fatty acids in *Pelophylax nigromaculatus* tadpoles. Environ Pollut. 2021;288: Article 117702. 34246997 10.1016/j.envpol.2021.117702

[15] Liu L, Li MZ, Yao MH, Yang TN, Tang YX, Li JL. Melatonin inhibits atrazine-induced mitochondrial impairment in cerebellum of mice: Modulation of cGAS-STING-NLRP3 axis-dependent cell pyroptosis. Sci Total Environ. 2024;912: Article 168924. 38036146 10.1016/j.scitotenv.2023.168924

[16] Shi G, Zhang C, Li G, Wang K, Cai Q, Huang M. Atrazine induces phagocytotic dysfunction of microglia depends on nucleocytoplasmic translocation of acetylated HMGB1. Ecotoxicol Environ Saf. 2023;252: Article 114583. 36736232 10.1016/j.ecoenv.2023.114583

[17] Stradtman SC, Freeman JL. Mechanisms of neurotoxicity associated with exposure to the herbicide atrazine. Toxics. 2021;9(9): Article 207. 34564358 10.3390/toxics9090207 PMC8473009

[18] Wirbisky SE, Freeman JL. Atrazine exposure and reproductive dysfunction through the hypothalamus-pituitary-gonadal (HPG) axis. Toxics. 2015;3(4):414–450. 28713818 10.3390/toxics3040414 PMC5507375

[19] Xiao YZ, Yang M, Xiao Y, Guo Q, Huang Y, Li CJ, Cai D, Luo XH. Reducing hypothalamic stem cell senescence protects against aging-associated physiological decline. Cell Metab. 2020;31(3):534–548.e5. 32004475 10.1016/j.cmet.2020.01.002

[20] Kanthasamy A, Jin H, Charli A, Vellareddy A, Kanthasamy A. Environmental neurotoxicant-induced dopaminergic neurodegeneration: A potential link to impaired neuroinflammatory mechanisms. Pharmacol Ther. 2019;197:61–82. 30677475 10.1016/j.pharmthera.2019.01.001 PMC6520143

[21] Shi YS, Yang TN, Wang YX, Ma XY, Liu S, Zhao Y, Li JL. Melatonin mitigates atrazine-induced renal tubular epithelial cell senescence by promoting parkin-mediated mitophagy. Research. 2024;7: Article 0378. 38766643 10.34133/research.0378 PMC11098712

[22] Zhu SY, Jiang JZ, Lin J, Liu L, Guo JY, Li JL. Lycopene ameliorates atrazine-induced spatial learning and memory impairments by inhibiting ferroptosis in the hippocampus of mice. Food Chem Toxicol. 2023;174: Article 113655. 36791905 10.1016/j.fct.2023.113655

[23] Zhang Y, Kim MS, Jia B, Yan J, Zuniga-Hertz JP, Han C, Cai D. Hypothalamic stem cells control ageing speed partly through exosomal miRNAs. Nature. 2017;548(7665):52–57. 28746310 10.1038/nature23282 PMC5999038

[24] Navarro Negredo P, Yeo RW, Brunet A. Aging and rejuvenation of neural stem cells and their niches. Cell Stem Cell. 2020;27(2):202–223. 32726579 10.1016/j.stem.2020.07.002 PMC7415725

[25] Zhao Y, Zhang H, Cui JG, Wang JX, Chen MS, Wang HR, Li XN, Li JL. Ferroptosis is critical for phthalates driving the blood-testis barrier dysfunction via targeting transferrin receptor. Redox Biol. 2023;59: Article 102584. 36580806 10.1016/j.redox.2022.102584 PMC9813583

[26] Ikeji CN, Adedara IA, Farombi EO. Dietary myricetin assuages atrazine-mediated hypothalamic-pituitary-testicular axis dysfunction in rats. Environ Sci Pollut Res Int. 2023;30(6):15655–15670. 36169847 10.1007/s11356-022-23033-5

[27] Wang J, He X, Meng H, Li Y, Dmitriev P, Tian F, Page JC, Lu QR, He Z. Robust myelination of regenerated axons induced by combined manipulations of GPR17 and microglia. Neuron. 2020;108(5):876–886.e4. 33108748 10.1016/j.neuron.2020.09.016 PMC7736523

[28] Mu W, Li S, Xu J, Guo X, Wu H, Chen Z, Qiao L, Helfer G, Lu F, Liu C, et al. Hypothalamic Rax^+^ tanycytes contribute to tissue repair and tumorigenesis upon oncogene activation in mice. Nat Commun. 2021;12(1): Article 2288. 33863883 10.1038/s41467-021-22640-z PMC8052410

[29] Wang C, Zhang H, Fan J, Li Q, Guo R, Pan J, Liu Y, Peng J, Zhu Q, Feng Y, et al. Inhibition of integrated stress response protects against lipid-induced senescence in hypothalamic neural stem cells in adamantinomatous craniopharyngioma. Neuro-Oncology. 2023;25(4):720–732. 36454228 10.1093/neuonc/noac261 PMC10076952

[30] Costa-Mattioli M, Walter P. The integrated stress response: From mechanism to disease. Science. 2020;368(6489): Article eaat5314. 32327570 10.1126/science.aat5314 PMC8997189

[31] GBD 2019 Dementia Forecasting Collaborators. Estimation of the global prevalence of dementia in 2019 and forecasted prevalence in 2050: An analysis for the Global Burden of Disease Study 2019. Lancet Public Health. 2022;7(2):e105–e125. 34998485 10.1016/S2468-2667(21)00249-8 PMC8810394

[32] Fan Y, Winanto NSY, Ng SY. Replacing what’s lost: A new era of stem cell therapy for Parkinson’s disease. Transl Neurodegener. 2020;9: Article 2. 10.1186/s40035-019-0180-x PMC6945567 31911835

[33] Graziano N, McGuire MJ, Roberson A, Adams C, Jiang H, Blute N. 2004 National Atrazine Occurrence Monitoring Program using the Abraxis ELISA method. Environ Sci Technol. 2006;40(4):1163–1171. 16572770 10.1021/es051586y

[34] Yang TN, Wang YX, Jian PA, Ma XY, Ren YF, Huang NN, Li XN, Li JL. Rab8a is a key target that melatonin prevents lipid disorder from atrazine. *J Agric Food Chem*. 2024;**72**(42):23511–23519. 10.1021/acs.jafc.4c07006 39382334

[35] Wiseman RL, Mesgarzadeh JS, Hendershot LM. Reshaping endoplasmic reticulum quality control through the unfolded protein response. Mol Cell. 2022;82(8):1477–1491. 35452616 10.1016/j.molcel.2022.03.025 PMC9038009

[36] Rai M, Curley M, Coleman Z, Demontis F. Contribution of proteases to the hallmarks of aging and to age-related neurodegeneration. Aging Cell. 2022;21(5): Article e13603. 35349763 10.1111/acel.13603 PMC9124314

[37] Chou HY, Liu LH, Chen CY, Lin IF, Ali D, Yueh-Luen Lee A, David Wang HM. Bifunctional mechanisms of autophagy and apoptosis regulations in melanoma from *Bacillus subtilis* natto fermentation extract. Food Chem Toxicol. 2021;150: Article 112020. 33513408 10.1016/j.fct.2021.112020

[38] How CM, Li SW, Liao VH. Chronic exposure to triadimenol at environmentally relevant concentration adversely affects aging biomarkers in *Caenorhabditis elegans* associated with insulin/IGF-1 signaling pathway. Sci Total Environ. 2018;640–641:485–492. 10.1016/j.scitotenv.2018.05.314 29864662

[39] Zhou W, Tong D, Tian D, Yu Y, Huang L, Zhang W, Yu Y, Lu L, Zhang X, Pan W, et al. Exposure to polystyrene nanoplastics led to learning and memory deficits in zebrafish by inducing oxidative damage and aggravating brain aging. Adv Healthc Mater. 2023;12(29): Article e2301799. 37611966 10.1002/adhm.202301799

[40] Ma K, Wu HY, Wang SY, Li BX. The Keap1/Nrf2-ARE signaling pathway is involved in atrazine induced dopaminergic neurons degeneration via microglia activation. Ecotoxicol Environ Saf. 2021;226: Article 112862. 34624533 10.1016/j.ecoenv.2021.112862

[41] Masaki Y, Izumi Y, Matsumura A, Akaike A, Kume T. Protective effect of Nrf2-ARE activator isolated from green perilla leaves on dopaminergic neuronal loss in a Parkinson’s disease model. Eur J Pharmacol. 2017;798:26–34. 28167258 10.1016/j.ejphar.2017.02.005

[42] Li J, Li X, Bi H, Li B. The MEK/ERK/CREB signaling pathway is involved in atrazine induced hippocampal neurotoxicity in Sprague Dawley rats. Ecotoxicol Environ Saf. 2019;170:673–681. 30580161 10.1016/j.ecoenv.2018.12.038

[43] Wang CL, Ohkubo R, Mu WC, Chen W, Fan JL, Song Z, Maruichi A, Sudmant PH, Pisco AO, Dubal DB, et al. The mitochondrial unfolded protein response regulates hippocampal neural stem cell aging. Cell Metab. 2023;35(6):996–1008e7. 37146607 10.1016/j.cmet.2023.04.012 PMC10330239

[44] Santos MFD, Roxo C, Sola S. Oxidative-signaling in neural stem cell-mediated plasticity: Implications for neurodegenerative diseases. Antioxidants. 2021;10(7): Article 1088. 34356321 10.3390/antiox10071088 PMC8301193

[45] Keele GR, Zhang JG, Szpyt J, Korstanje R, Gygi SP, Churchill GA, Schweppe DK. Global and tissue-specific aging effects on murine proteomes. Cell Rep. 2023;42(7): Article 112715. 37405913 10.1016/j.celrep.2023.112715 PMC10588767

[46] Derisbourg MJ, Hartman MD, Denzel MS. Perspective: Modulating the integrated stress response to slow aging and ameliorate age-related pathology. Nat Aging. 2021;1(9):760–768. 35146440 10.1038/s43587-021-00112-9 PMC7612338

[47] Krukowski K, Nolan A, Frias ES, Boone M, Ureta G, Grue K, Paladini MS, Elizarraras E, Delgado L, Bernales S, et al. Small molecule cognitive enhancer reverses age-related memory decline in mice. elife. 2020;9: Article e62048. 33258451 10.7554/eLife.62048 PMC7721440

[48] Urban N, Blomfield IM, Guillemot F. Quiescence of adult mammalian neural stem cells: A highly regulated rest. Neuron. 2019;104(5):834–848. 31805262 10.1016/j.neuron.2019.09.026

[49] de Morree A, Rando TA. Regulation of adult stem cell quiescence and its functions in the maintenance of tissue integrity. Nat Rev Mol Cell Biol. 2023;24(5):334–354. 36922629 10.1038/s41580-022-00568-6 PMC10725182

[50] Huang L, Zhang L. Neural stem cell therapies and hypoxic-ischemic brain injury. Prog Neurobiol. 2019;173:1–17. 29758244 10.1016/j.pneurobio.2018.05.004 PMC6249121

[51] Du Q, Deng R, Li W, Zhang D, Tsoi B, Shen J. Baoyuan Capsule promotes neurogenesis and neurological functional recovery through improving mitochondrial function and modulating PI3K/Akt signaling pathway. Phytomedicine. 2021;93: Article 153795. 34735905 10.1016/j.phymed.2021.153795

[52] Wang Y, Lafon PA, Salvador-Prince L, Gines AR, Trousse F, Torrent J, Prevostel C, Crozet C, Liu J, Perrier V. Prenatal exposure to low doses of fungicides corrupts neurogenesis in neonates. Environ Res. 2021;195: Article 110829. 33548298 10.1016/j.envres.2021.110829

[53] Colle D, Farina M, Ceccatelli S, Raciti M. Paraquat and Maneb exposure alters rat neural stem cell proliferation by inducing oxidative stress: New insights on pesticide-induced neurodevelopmental toxicity. Neurotox Res. 2018;34(4):820–833. 29859004 10.1007/s12640-018-9916-0

[54] Zhu SY, Li XN, Zhao Y, Dai XY, Guo JY, Li JL. Lycopene ameliorate atrazine-induced oxidative damage in the B cell zone via targeting the miR-27a-3p/Foxo1 Axis. J Agric Food Chem. 2022;70(39):12502–12512. 36134885 10.1021/acs.jafc.2c05103

[55] Victor-Costa AB, Bandeira SM, Oliveira AG, Mahecha GAB, Oliveira CA. Changes in testicular morphology and steroidogenesis in adult rats exposed to atrazine. Reprod Toxicol. 2010;29(3):323–331. 20045047 10.1016/j.reprotox.2009.12.006

[56] Zhu SY, Guo JY, Li JY, Dai XY, Li XN, Li JL. Lycopene ameliorates atrazine-induced pyroptosis in spleen by suppressing the ox-mtDNA/Nlrp3 inflammasome pathway. Food Funct. 2022;13(6):3551–3560. 35260874 10.1039/d1fo02857j

[57] Li B, Jiang Y, Wang T, He X, Ma L, Li B, Li Y. Effect of atrazine on accumulation of iron via the iron transport proteins in the midbrain of SD rats. Sci Total Environ. 2021;780: Article 146666. 34030342 10.1016/j.scitotenv.2021.146666

[58] Sharp MM, Page A, Morris A, Weller RO, Carare RO. Quantitative assessment of cerebral basement membranes using electron microscopy. Methods Mol Biol. 2017;1559:367–375. 28063057 10.1007/978-1-4939-6786-5_25

[59] Zhao Y, Hu ZY, Lou M, Jiang FW, Huang YF, Chen MS, Wang JX, Liu S, Shi YS, Zhu HM, et al. AQP1 deficiency drives phthalate-induced epithelial barrier disruption through intestinal inflammation. J Agric Food Chem. 2024;72(27):15334–15344. 38916549 10.1021/acs.jafc.4c03764

[60] Cui T, Jiang W, Yang F, Luo J, Hu R, Cao H, Hu G, Zhang C. Molybdenum and cadmium co-induce hypothalamus toxicity in ducks via disturbing Nrf2-mediated defense response and triggering mitophagy. Ecotoxicol Environ Saf. 2021;228: Article 113022. 34844167 10.1016/j.ecoenv.2021.113022

[61] Chen J, Xu XW, Kang JX, Zhao BC, Xu YR, Li JL. Metasilicate-based alkaline mineral water confers diarrhea resistance in maternally separated piglets via the microbiota-gut interaction. Pharmacol Res. 2023;187: Article 106580. 36436708 10.1016/j.phrs.2022.106580

[62] Chen J, Dai X, Xing C, Zhang Y, Cao H, Hu G, Guo X, Gao X, Liu P, Yang F. Cooperative application of transcriptomics and ceRNA hypothesis: lncRNA-00742/miR-116 targets CD74 to mediate vanadium-induced mitochondrial apoptosis in duck liver. *J Hazard Mater*. 2024;480:Article 135904. 10.1016/j.jhazmat.2024.135904 39303616

[63] Chen J, Dai XY, Zhao BC, Xu XW, Kang JX, Xu YR, Li JL. Role of the GLP2-Wnt1 axis in silicon-rich alkaline mineral water maintaining intestinal epithelium regeneration in piglets under early-life stress. Cell Mol Life Sci. 2024;81(1): Article 126. 38470510 10.1007/s00018-024-05162-x PMC10933158

[64] Qiao N, Dai X, Chen J, Cao H, Hu G, Guo X, Liu P, Xing C, Yang F. Single nucleus RNA sequencing reveals cellular and molecular responses to vanadium exposure in duck kidneys. *J Hazard Mater*. 2024;**480**:Article 136492. 10.1016/j.jhazmat.2024.136492 39541890

[65] Hu ZY, Yang SJ, Chang YH, Wang XQ, Liu RQ, Jiang FW, Chen MS, Wang JX, Liu S, Zhu HM, et al. AHR activation relieves deoxynivalenol-induced disruption of porcine intestinal epithelial barrier functions. J Hazard Mater. 2024;480: Article 136095. 39395393 10.1016/j.jhazmat.2024.136095

[66] Chen J, Zhao BC, Dai XY, Xu YR, Kang JX, Li JL. Drinking alkaline mineral water confers diarrhea resistance in maternally separated piglets by maintaining intestinal epithelial regeneration via the brain-microbe-gut axis. J Adv Res. 2023;52:29–43. 36539076 10.1016/j.jare.2022.12.008 PMC10555785

[67] Livak KJ, Schmittgen TD. Analysis of relative gene expression data using real-time quantitative PCR and the 2^−ΔΔ*C*T^ method. Methods. 2001;25(4):402–408. 11846609 10.1006/meth.2001.1262

