# Erratum to “A New Insight into the Mechanism of Atrazine-Induced Neurotoxicity: Triggering Neural Stem Cell Senescence by Activating the Integrated Stress Response Pathway”

**DOI:** 10.34133/research.0641

**Published:** 2025-03-25

**Authors:** Jian Chen, Xue-Yan Dai, Kanwar K. Malhi, Xiang-Wen Xu, Yi-Xi Tang, Xiao-Wei Li, Jin-Long Li

**Affiliations:** ^1^ College of Veterinary Medicine, Northeast Agricultural University, Harbin 150030, P.R. China.; ^2^ Jiangxi Provincial Key Laboratory for Animal Health, Institute of Animal Population Health, College of Animal Science and Technology, Jiangxi Agricultural University, Nanchang 330045, P.R. China.; ^3^ Key Laboratory of the Provincial Education Department of Heilongjiang for Common Animal Disease Prevention and Treatment, Northeast Agricultural University, Harbin 150030, P.R. China.

In the Research Article “A New Insight into the Mechanism of Atrazine-Induced Neurotoxicity: Triggering Neural Stem Cell Senescence by Activating the Integrated Stress Response Pathway,” an error needs to be corrected in Figure 5 [1].

**Fig. 5.**
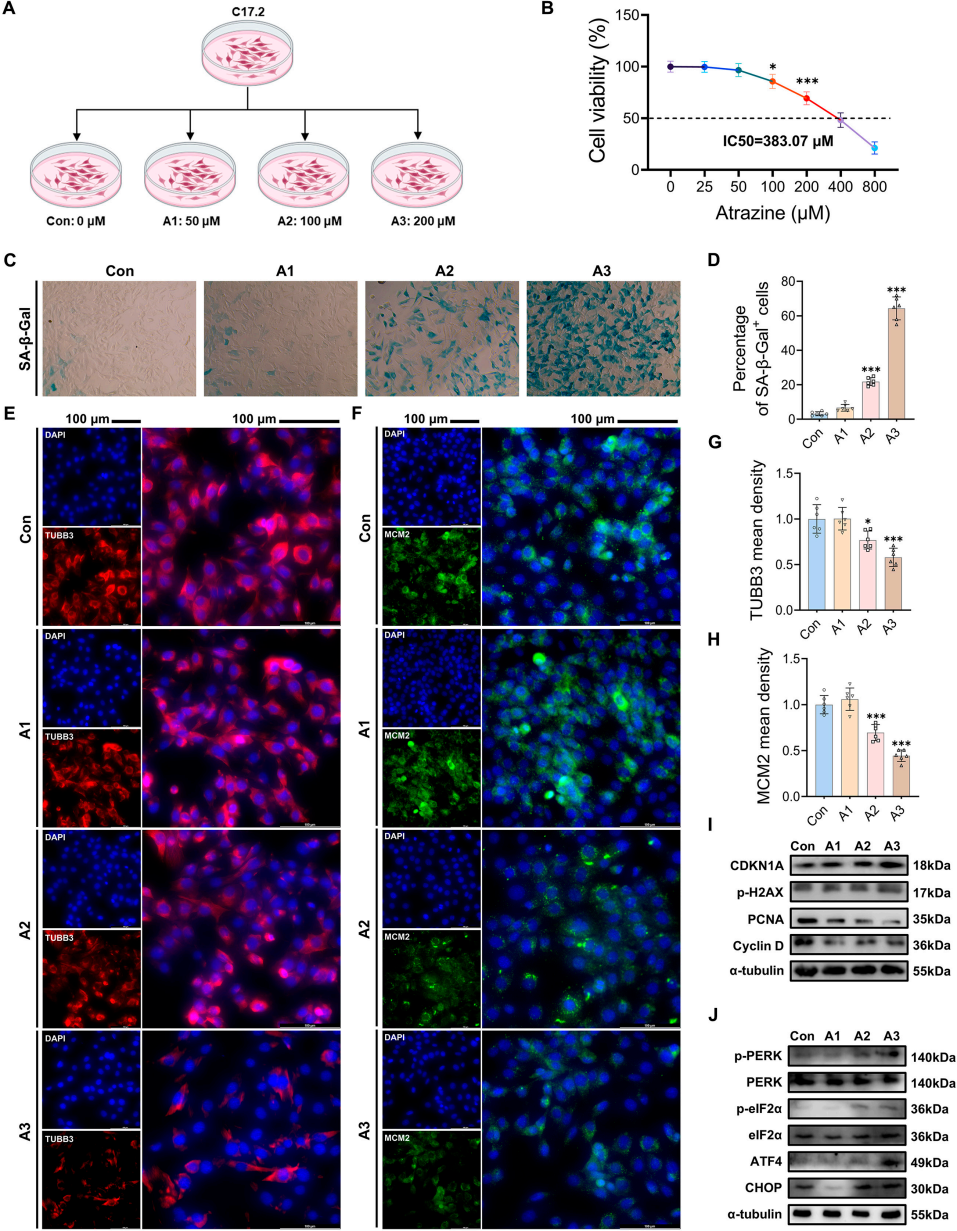
Atrazine exposure induced senescence and ISR signal activation in C17.2 NSCs. (A) C17.2 NSCs were treated with atrazine. (B) Cell viability across various concentrations of atrazine treatment. (C) Representative image of C17.2 NSCs by senescence-associated β-galactosidase (SA-β-Gal) staining. Magnification: ×400. (D) Statistical analysis of percentage of SA-β-Gal^+^ cells. (E) Representative images of 4′,6-diamidino-2-phenylindole (DAPI) and TUBB3 staining. (F) Representative images of DAPI and MCM2 staining; scale bars, 100 μm. (G and H) Statistical analysis of (G) TUBB3 and (H) MCM2 mean density. (I) Western blotting measurements of the protein levels of cyclin-dependent kinase inhibitor 1A (CDKN1A), phosphorylated histone H2A variant Xp-H2AX (p-H2AX), proliferating cell nuclear antigen (PCNA), and cyclin D in C17.2 NSCs. (J) Western blotting measurements of the protein levels of the ISR signaling pathway in C17.2 NSCs. The data are presented as mean ± SD. Statistical analysis was performed using one-way ANOVA for multiple group comparisons followed by Tukey’s post hoc pairwise comparison. *P < 0.05 and ***P < 0.001 vs. the Con group.

During a post-publication review, the authors identified an inadvertent error in Figure 5. Specifically, in panel F, the image for the A2 treatment group was incorrectly included. This was due to an error during the figure assembly, where an incorrect image was mistakenly inserted into the PowerPoint file. Additionally, the position-marking layer was not removed, which resulted in the incorrect image being published.

The authors wish to emphasize that this error does not affect the data analysis, results, or conclusions of our study. The figure has now been corrected in the original publication, and it is also presented below.
