# Supplementary material for: A New Insight into the Mechanism of Atrazine-Induced Neurotoxicity: Triggering Neural Stem Cell Senescence by Activating the Integrated Stress Response Pathway
Source: Research (Wash D C). 2024 Dec 13;7:0547. doi: 10.34133/research.0547 (PMC11638487; doi:10.34133/research.0547)
Supplement: Supplementary 1 — Figs. S1 to S3 Tables S1 and S2 [file research.0547.f1.docx]

***Supporting Information***

**Title page**

**A New Insight into the Mechanism of** **Atrazine-Induced Neurotoxicity: Triggering Neural Stem Cell Senescence by Activating Integrated Stress Response Pathway**

Jian Chen^1^^†^, Xue-Yan Dai^2†^, Kanwar K. Malhi^1^, Xiang-Wen Xu^1^, Yi-Xi Tang^1^, Xiao-Wei Li^1^, Jin-Long Li^1,3*^

^1^College of Veterinary Medicine, Northeast Agricultural University, Harbin 150030, P.R. China

^2^Jiangxi Provincial Key Laboratory for Animal Health, Institute of Animal Population Health, College of Animal Science and Technology, Jiangxi Agricultural University, No. 1101 Zhimin Avenue, Nanchang 330045, P.R. China

^3^Key Laboratory of the Provincial Education Department of Heilongjiang for Common Animal Disease Prevention and Treatment, Northeast Agricultural University, Harbin 150030, P.R. China

^†^These authors contributed equally.

***Corresponding author**

**Jin-Long Li**

Address: College of Veterinary Medicine, Northeast Agricultural University, Harbin, 150030, P. R. China

Tel: +86 45 155190407; fax: +86 451 55190407. E-mail address: Jinlongli@neau.edu.cn (J.L. Li)

**Supplemental Contents**

Materials and methods of RNA-Seq

Tables S1 to S2

Figs. S1 to S3

**Materials and methods of RNA-Seq**

*Sample collection and preparation*

- RNA degradation and contamination was monitored on 1% agarose gels.
- RNA purity was checked using the NanoPhotometer® spectrophotometer (IMPLEN, CA, USA).
- RNA integrity was assessed using the RNA Nano 6000 Assay Kit of the Bioanalyzer 2100 system (Agilent Technologies, CA, USA).

*Library preparation for RNA-Seq sequencing*

- A total amount of 1 µg RNA per sample was used as input material for the RNA sample preparations. Sequencing libraries were generated using NEBNext® UltraTM RNA Library Prep Kit for Illumina® (NEB, USA) following manufacturer’s recommendations and index codes were added to attribute sequences to each sample.
- Briefly, mRNA was purified from total RNA using poly-T oligo-attached magnetic beads. Fragmentation was carried out using divalent cations under elevated temperature in NEBNext First Strand Synthesis Reaction Buffer(5X). First strand cDNA was synthesized using random hexamer primer and M-MuLV Reverse Transcriptase (RNase H-). Second strand cDNA synthesis was subsequently performed using DNA Polymerase I and RNase H. Remaining overhangs were converted into blunt ends via exonuclease/polymerase activities. After adenylation of 3’ ends of DNA fragments, NEBNext Adaptor with hairpin loop structure were ligated to prepare for hybridization. In order to select cDNA fragments of preferentially 250~300 bp in length, the library fragments were purified with AMPure XP system (Beckman Coulter, Beverly, USA). Then 3 µl USER Enzyme (NEB, USA) was used with size-selected, adaptor-ligated cDNA at 37°C for 15 min followed by 5 min at 95℃ before PCR. Then PCR was performed with Phusion High-Fidelity DNA polymerase, Universal PCR primers and Index (X) Primer. At last, PCR products were purified (AMPure XP system) and library quality was assessed on the Agilent Bioanalyzer 2100 system.

*Clustering and sequencing*

- The clustering of the index-coded samples was performed on a cBot Cluster. Generation System using TruSeq PE Cluster Kit v3-cBot-HS (Illumia) according to the manufacturer’s instructions. After cluster generation, the library preparations were sequenced on an Illumina Novaseq platform and 150 bp paired-end reads were generated.

*Data Analysis*

- Quality control: Raw data (raw reads) of fastq format were firstly processed through in-house perl scripts. In this step, clean data (clean reads) were obtained by removing reads containing adapter, reads containing ploy-N and low-quality reads from raw data. At the same time, Q20, Q30 and GC content the clean data were calculated. All the downstream analyses were based on the clean data with high quality.
- Reads mapping to the reference genome: Reference genome and gene model annotation files were downloaded from genome website directly. Index of the reference genome was built using Hisat2 v2.0.5 and paired-end clean reads were aligned to the reference genome using Hisat2 v2.0.5.
- Differential expression analysis: Differential expression analysis of two conditions/groups (two biological replicates per condition) was performed using the DESeq2 R package (1.16.1). DESeq2 provide statistical routines for determining differential expression in digital gene expression data using a model based on the negative binomial distribution. The resulting P-values were adjusted using the Benjamini and Hochberg’s approach for controlling the false discovery rate. Genes with an adjusted P-value <0.05 found by DESeq2 were assigned as differentially expressed.
- GSEA: the enrichment analysis was performed on these expressed data using GSEA software (V4.1.0). In this process, the KEGG gene sets (c2.cp.kegg.v7.0.symbols.gmt) was selected as the functional gene set, other parameters as the default settings. The pathway of gene enrichment with a normal p-value<0.05 and FDR q-value<0.25 has the significance of the statistics.

**Table S1.** **Antibodies used in this study**

| Antibody name | Host | Company | product category |
| --- | --- | --- | --- |
| Anti-MBP antibody | Rabbit | Proteintech | 10458-1-AP |
| Anti-MBP antibody | Mouse | Proteintech | 66003-1-Ig |
| Anti-NF antibody | Mouse | Proteintech | 60331-1-Ig |
| anti- MAP2 antibody | Rabbit | Affinity | AF4081 |
| anti-NeuN antibody | Mouse | Proteintech | 66836-1-Ig |
| anti-IBA-1 antibody | Rabbit | Abclonal | A19776 |
| anti-SOX2 antibody | Mouse | Abcam | ab79351 |
| anti-DCX antibody | Rabbit | Abclonal | A14611 |
| anti-TUBB3 antibody | Rabbit | Affinity | AF7000 |
| anti-MCM2 antibody | Rabbit | Affinity | AF0206 |
| anti-γ-H2AX antibody | Rabbit | Abclonal | A2082 |
| anti-Lamin B1 antibody | Rabbit | Bioss | bs-23709R |
| anti-p-PERK antibody | Rabbit | Abclonal | AP0886 |
| anti-PERK antibody | Rabbit | Abclonal | A18196 |
| anti-p-eIF2α antibody | Rabbit | Abclonal | AP0692 |
| anti- eIF2α antibody | Rabbit | Abclonal | A0764 |
| anti-ATF4 antibody | Rabbit | Affinity | DF6008 |
| anti-CHOP antibody | Rabbit | Abclonal | A20987 |
| anti-CDKN1A antibody | Rabbit | Abclonal | A1483 |
| anti-p-H2AX antibody | Rabbit | Abclonal | AP0687 |
| anti-PCNA antibody | Rabbit | Abclonal | A9909 |
| anti-Cyclin D antibody | Rabbit | Affinity | AF0931 |
| anti-α-tubulin antibody | Rabbit | Affinity | AF4651 |
| HRP-conjugated secondary antibody^-^ | Goat | Bioss | bs-40295G-HRP |
| Anti-Rabbit IgG H&L (Alexa Fluor® 488) | Goat | Abcam | ab150077 |
| Anti-Rabbit IgG H&L (Alexa Fluor® 594) | Goat | Abcam | ab150080 |
| Anti-Mouse IgG H&L (Alexa Fluor® 488) | Goat | Abcam | ab150113 |
| Anti-Mouse IgG H&L (Alexa Fluor® 594) | Goat | Abcam | ab150116 |

**Table S2. Primers used for qRT-PCR analysis**

| Gene name | Accession number | Primer and probe sequences (5′ to 3′) |
| --- | --- | --- |
| GAPDH1 | NM_001289726.2 | F: CAGGAGAGTGTTTCCTCGTCC  R: GATGGGCTTCCCGTTGATGA |
| GAPDH2 | NM_001289726.2 | F: ACCCTTAAGAGGGATGCTGC  R: CCCAATACGGCCAAATCCGT |
| MMP3 | NM_010809.3 | F: CAGTCCCTCTATGGAACTCCC  R: AGGGTGCTGACTGCATCAAA |
| CDKN2A | NM_001040654.1 | F: TGAATCTCCGCGAGGAAAGC  R: TGCCCATCATCATCACCTGAA |
| IL-6 | NM_001314054.1 | F: ACTTCACAAGTCGGAGGCTT  R: GTGACTCCAGCTTATCTCTTGGT |
| IL-8 | NM_011339.2 | F: CTAGGCATCTTCGTCCGTCC  R: CAGAAGCTTCATTGCCGGTG |
| CXCL1 | NM_011339.2 | F: CTAGGCATCTTCGTCCGTCC  R: CAGAAGCTTCATTGCCGGTG |


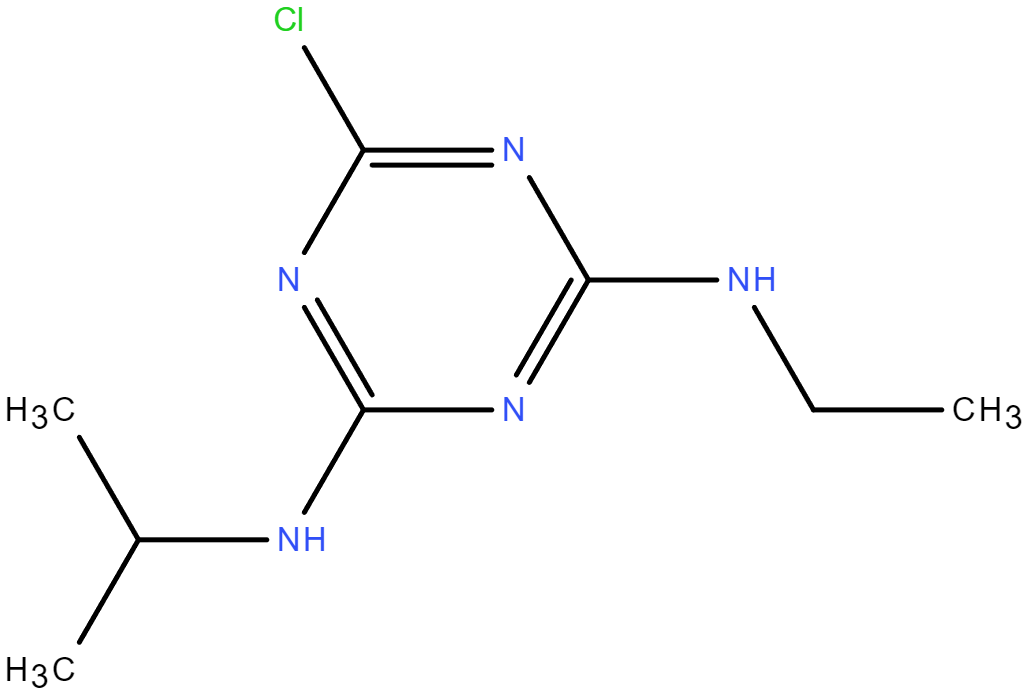


**Figure S1. The chemical structure of atrazine.**


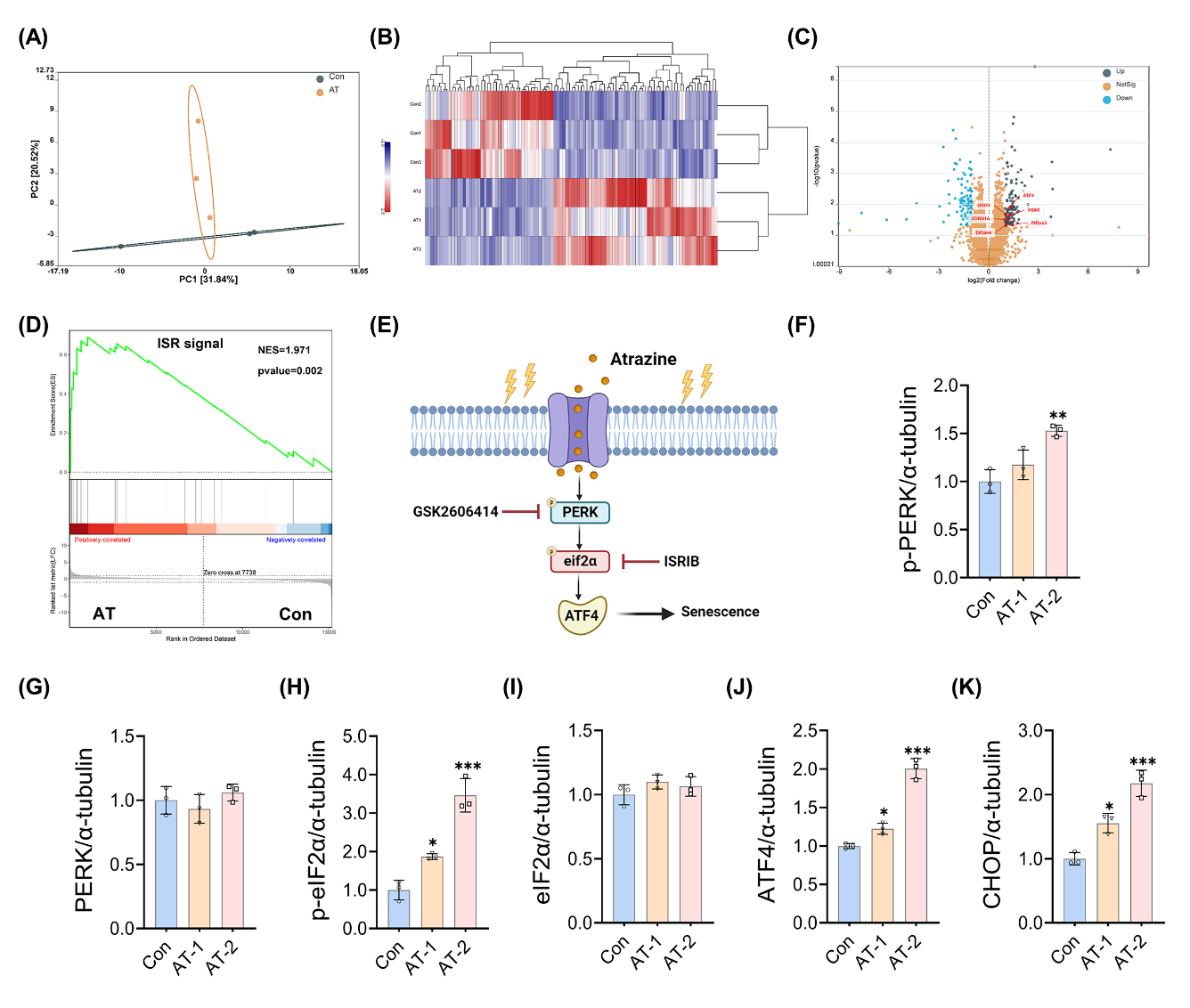


**Figure S2. Atrazine exposure induces DNA damage and senescence of the recruited HtNSCs by activating the ISR signaling.** (A) Principal component analysis (PCA) of hypothalamus from the Con and the AT treatment groups. (B) Gene expression profiling via RNA-sequencing shows differentially expressed genes (DEGs) between Con and AT groups based on hierarchization clustering. (C) Volcano plot showing the down-regulated and up-regulated DEGs of two groups. (D) Gene set enrichment analysis (GSEA) indicating significant enrichment for the ISR signaling pathway in hypothalamus after atrazine exposure. (E) Schematic diagram showing ISR signaling pathway and ISR inhibitors (ISRIB and GSK2606414) target. (F-K) Statistical analysis of (F) p-PERK, (G) PERK, (H) p-eiF2α, (I) eiF2α, (J) ATF4 and (K) CHOP protein levels.


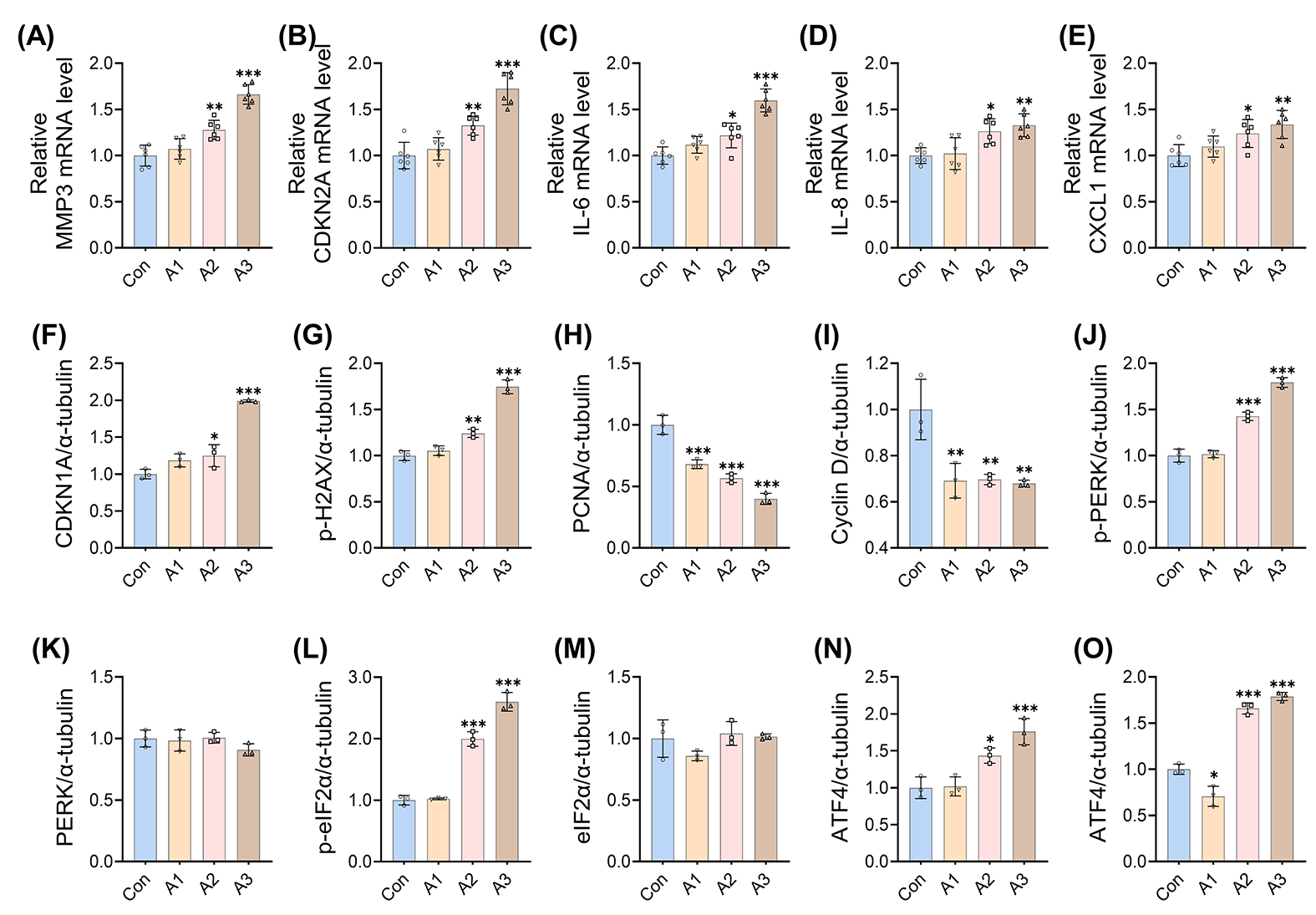


**Figure S3.** **Atrazine exposure induces senescence and integrated stress response (ISR) signaling activation in C17.2 neural stem cells (NSCs).** (A-E) Statistical analysis of (A) MMP3, (B) CDKN2A, (C) IL-6, (D) IL-8, and (E) CXCL1 relative mRNA levels. (F-O) Statistical analysis of (F) CDKN1A, (G) p-H2AX, (H) PCNA, (I) Cyclin D, (J) p-PERK, (K) PERK, (L) p-eiF2α, (M) eiF2α, (N) ATF4 and (O) CHOP protein levels. The data are presented as the means ± SDs. Statistical analysis was performed with one-way ANOVA for multiple group comparisons followed by Tukey’s post hoc pairwise comparison. ^*^*P*<0.05, ^**^*P*<0.01, and ^***^*P*<0.001 vs. the Con group.


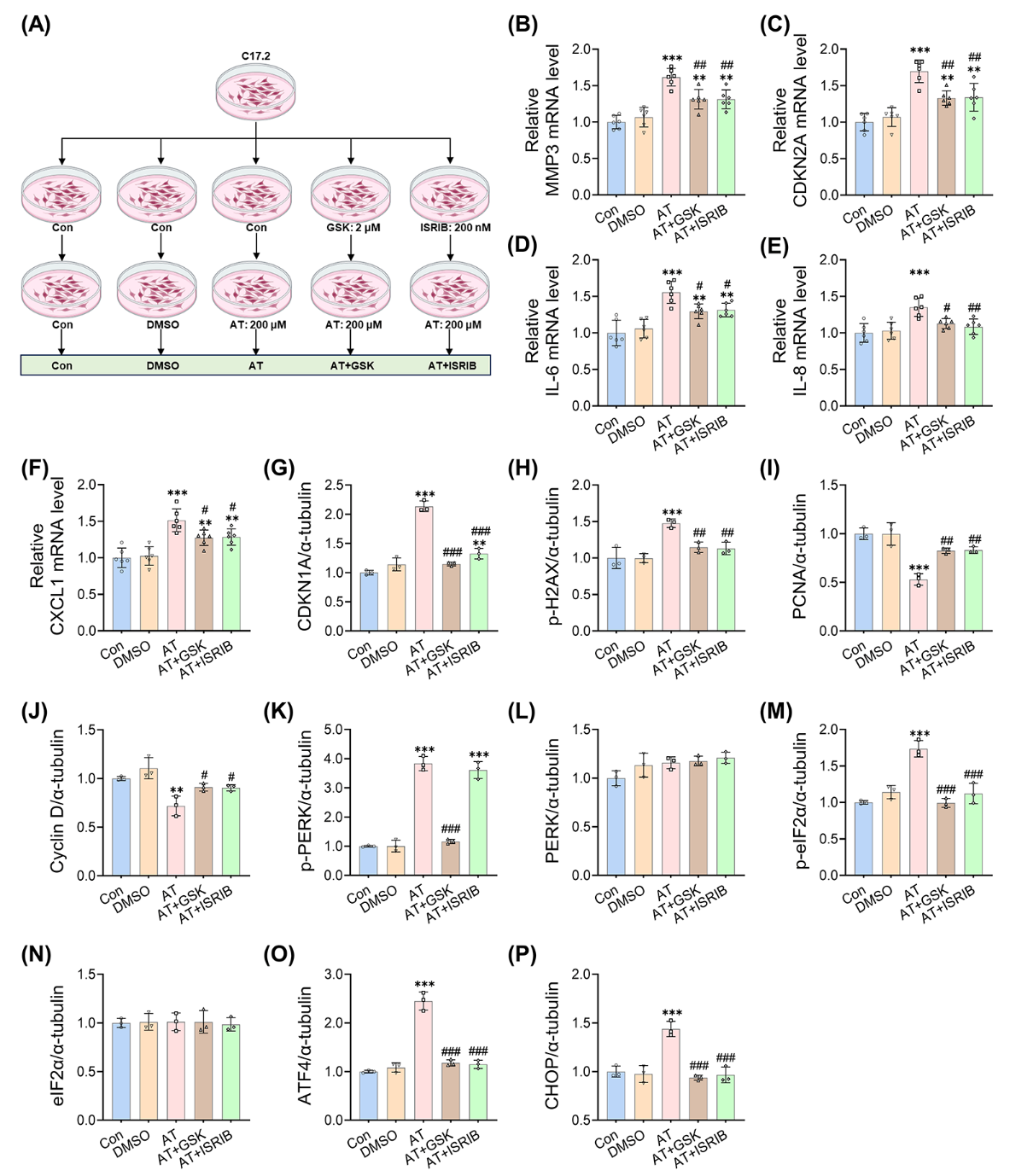


**Fig. S4.** **Inhibition of integrated stress response (ISR) signaling alleviates atrazine (AT)-induced senescence of C17.2 neural stem cells (NSCs).** (A) C17.2 NSCs were treated with AT, DMSO, GSK, and ISRIB. (B-F) Statistical analysis of (B) MMP3, (C) CDKN2A, (D) IL-6, (E) IL-8, and (F) CXCL1 relative mRNA levels. (G-P) Statistical analysis of (G) CDKN1A, (H) p-H2AX, (I) PCNA, (J) Cyclin D, (K) p-PERK, (L) PERK, (M) p-eiF2α, (N) eiF2α, (O) ATF4 and (P) CHOP protein levels. The data are presented as the means ± SDs. Statistical analysis was performed with one-way ANOVA for multiple group comparisons followed by Tukey’s post hoc pairwise comparison. ^*^*P*<0.05, ^**^*P*<0.01, and ^***^*P*<0.001 vs. the Con group. ^#^*P*<0.05, ^##^*P*<0.01, and ^###^*P*<0.001 vs. the AT group.
